# Continuous wavelet based transfer function analysis of cerebral autoregulation dynamics for neuromonitoring using near-infrared spectroscopy

**DOI:** 10.3389/fphys.2025.1616125

**Published:** 2025-06-18

**Authors:** Marcus Thudium, Evgeniya Kornilov, Stefan Moestl, Fabian Hoffmann, Alex Hoff, Surat Kulapatana, Vasile Urechie, Maximilian Oremek, Stefano Rigo, Karsten Heusser, Italo Biaggioni, Jens Tank, André Diedrich

**Affiliations:** ^1^ University Hospital Bonn, Department of Anesthesiology, Bonn, Germany; ^2^ Weizmann Institute of Science, Department of Neurobiology, Rehovot, Israel; ^3^ Department of Anesthesiology, Rabin Medical Center, Beilinson Hospital, Petach Tikvah, Israel; ^4^ German Aerospace Center, Institute of Aerospace Medicine, Cologne, Germany; ^5^ Department of Cardiology, University Hospital Cologne, Cologne, Germany; ^6^ Autonomic Dysfunction Center, Division of Clinical Pharmacology, Department of Medicine, Vanderbilt University Medical Center, Nashville, TN, United States; ^7^ Department of Biomedical Engineering, Vanderbilt University, Nashville, TN, United States; ^8^ Department of Physiology, Faculty of Medicine Siriraj Hospital, Mahidol University, Bangkok, Thailand; ^9^ Clinical Research Unit, myDoctorAngel Sagl, Bioggio, Switzerland; ^10^ Humanitas University, Department of Biomedical Sciences, Pieve Emanuele, Italy; ^11^ Humanitas Clinical and Research Center- IRCCS, Via Alessandro Manzoni, Rozzano, Italy

**Keywords:** cerebral autoregulation, transfer function analysis, wavelet, lower body negative pressure, lower body positive pressure

## Abstract

**Introduction:**

Near-infrared spectroscopy is now a popular method in neuromonitoring. Derived parameters like cerebral oxygenation index or Fast Fourier Transform based coherence estimates between blood pressure and cerebral blood flow have their limitations of use for stationary data and low time resolution. Wavelet transfer function analysis can be employed to estimate coherence, gain and phase relationship between two signals without these restrictions.

**Methods:**

We aimed to extend the previously described Grinsted wavelet package with rectified bias of power and transfer function gain estimation of cerebral autoregulation assessment. The algorithm was validated in simulated signals and data of five healthy male subjects undergoing a protocol to produce large changes in hemodynamics by using lower body positive pressure (LBNP) with mild hypoxia and lower body negative pressure (LBNP). We intended to compare wavelet-based observations with FFT-based estimates.

**Results:**

We found good agreement between wavelet and FFT-based coherence and gain of cerebral tissue oxygenation index, in Bland Altman Plot and linear correlations for repeated measurement especially in the low frequency range (0.04 -0.15 Hz, coherence: r = 0.69, p < 0.001, gain: r = 0.74, p = 0.001), but was less in the very low frequency range (≤0.04 Hz, coherence: r = 0.65, p < 0.001, gain: r = 0.66, p < 0.001) and high frequency range (0.15-0.4 Hz, coherence: r = 0.39, p < 0.001, gain: r = 0.71, p = 0.001). FFT-based coherence was smaller than wavelet estimates for values <0.5.

**Discussion:**

We demonstrated good agreement in power, coherence, transfer function gain estimates between FFT-based method and our modified wavelet method. This was confirmed in simulated data and in healthy subjects undergoing LBNP and LBPP with hypoxia. Near-infrared spectroscopy-derived wavelet transform could be useful for exploring cerebral autoregulation dynamics, especially in non-stationary data.

## Introduction

Cerebral autoregulation is an important characteristic of the cerebral vasculature to maintain adequate cerebral perfusion even in the presence of changing arterial driving pressure. The concept, originally introduced by Lassen, suggests a stable plateau on the autoregulation curve beyond which cerebral blood flow becomes pressure passive, potentially leading to damage by over- or underperfusion of the brain (Lassen, 1959; Lassen and Christensen, 1976). Apart from exceeding the limits of cerebral autoregulation, certain diseases can also affect autoregulation integrity with an increased risk of cerebrovascular events leading to unfavorable outcome (Thudium et al., 2024; Soehle et al., 2004).

Several methods of data integration and processing with other cardiovascular parameters have been proposed to allow insights into cerebral autoregulation properties. With an increased use of near-infrared spectroscopy (NIRS) as a surrogate parameter for cerebral blood flow, time domain parameters like moving correlation coefficients have been employed to describe the linear relationship between NIRS and mean arterial blood pressure (MBP). A cerebral oxygenation index (COx) has been introduced implementing a moving Pearson correlation coefficient between NIRS saturation and arterial blood pressure similar to the original mean velocity index (Mx) which is based on the correlation between transcranial Doppler sonography and MBP. These indices have been used on many occasions, and have even been used in clinical settings (Soehle et al., 2004; Brady et al., 2010; Joshi et al., 2012; Laflam et al., 2015). Time domain methods can observe changes over time but are limited to observe changes in a certain frequency ranges like rapid or slower fluctuations. Frequency domain methods based on Fast Fourier Transform (FFT) describe the phase relationship, gain and the transfer function between MBP as an input and NIRS as an output signal. These methods can be used to describe autoregulation properties in a wide range of frequencies. Coherence analyses in the frequency-domain have been utilized to estimate autoregulatory properties especially in the very low frequency range (VLF: ≤0.04 Hz) (Brady et al., 2010). However, FFT based coherence analysis requires stationary data and its time-frequency resolution is not optimized.

Continuous wavelet transform (CWT) and wavelet coherence have been introduced in cerebral autoregulation assessment (Tian et al., 2016; Cui et al., 2014). The wavelet method convolutes a time-domain signal with a selected scaled and sliding mother wavelet, in contrast to fixed time windows of the FFT-based method. The scaled wavelet alleviates the time resolution limitation, while the sliding wavelet overcomes the request of stationarity (Grinsted et al., 2004). Tian et al. applied continuous wavelet coherence in pediatric patients successfully. They demonstrated that in-phase and anti-phase coherences are related to worse clinical outcome (Tian et al., 2016). However, observed changes were over long periods from 80 min to 2.5 h. Cui et al. found age related differences in coherence and phase between elderly and young subjects using wavelet coherence analysis in higher frequency range (Cui et al., 2014). This demonstrates the importance to study also faster oscillations to characterize dynamic cerebral autoregulation.

More recent concepts of cerebral autoregulation suggest a rather mild sloped region instead of a completely flat plateau in the autoregulation curve as originally proposed by Lassen (Lassen, 1959; Brassard et al., 2021). This may lead to a difficult distinction between intact and impaired cerebral autoregulation when using COx or Mx calculations using correlation coefficients since a certain degree of correlation does not always imply disturbed autoregulation. Additionally, there is local and neural mediated vasomotion causing periodicities with low frequency oscillations (LF: 0.04 – 0.15 Hz, periodicities between 10 and 20 s) in the cardiovascular system which have not been studied fully as a separate entity in dynamic cerebral blood flow. Moving correlation coefficients process the signal over the whole frequency range and cannot distinguish LF oscillations from other changes in hemodynamics. FFT based coherence analysis allows to study LF oscillations but cannot be applied to short non-stationary data. The continuous wavelet transform allows to estimate coherence with optimal frequency and temporal resolution. Using only coherence cannot estimate the damping magnitude of external blood pressure disturbances on cerebral blood circulation. In this context, transfer function analysis can be employed to estimate gain and phase relationship between these two signals. The wavelet package by Grinsted introduced coherence analysis of geophysical time series has not implemented and validated transfer function estimates. Additionally, distorted wavelet spectral power has been reported and needs to be corrected in the algorithm (Grinsted et al., 2004; Liu et al., 2007).

Our objective was to extend the Grinsted wavelet package with a transfer function analysis with rectified estimation of power and validated transfer function gain applicable for assessment of dynamic cerebral autoregulation. We hypothesized that wavelet transfer function gain can be used to detect dynamic hemodynamic changes in the cerebral circulation under extreme conditions. We intended to test if 1) the modified wavelet transfer function delivers similar results compared to FFT-based method 2) cerebral tissue oxygenation by near infrared spectroscopy (NIRS) and cerebral blood velocity by transcranial Doppler (TCD) results reflect impaired cerebral autoregulation as compared to baseline measurements. To be suitable for application in dynamic clinical settings, we intended to focus especially on the LF range which is under the control of autonomic nervous system.

In order to test the validity of the wavelet package in conjunction with NIRS monitoring, it was necessary to observe a wide range of hemodynamic states, possibly exceeding the limits of the autoregulatory plateau as originally described by Lassen (Lassen, 1959). Therefore, we intended to use incremental lower body negative pressure (LBNP) to reduce cardiac preload. For hyperdynamic states, we aimed to use increased cardiac preload with incremental lower body positive pressure (LBPP) with added mild hypoxia on the last pressure level. Hypoxia has previously been reported to be associated with impaired cerebral autoregulation (Ainslie et al., 2008).

The aim of this pilot study was to validate wavelet transfer function algorithm with surrogate data and during a wide range of cerebral blood flow disturbances, induced by lower body negative or positive pressure and additional hypoxia, in a homogenous group of young healthy male subjects with intact cerebral autoregulation in order to receive uniform cerebrovascular responses to adequately observe differences between intact and impaired cerebral autoregulation.

## Materials and methods

LBNP and LBPP and additional hypoxia protocols were used to modulate blood pressure fluctuations and cerebrovascular response in healthy subjects. The protocol was approved by the ethics committee of the chamber of physicians North-Rhine, Duesseldorf, Germany (Chair: Prof. K. Racké, No 2018246). A signed informed consent was obtained for all subjects before participation.

### Enrollment

Subjects were enrolled after medical examination and confirmation of healthy status. Inclusion criteria were healthy, male subjects of age 18-30 years. Exclusion criteria were arterial hypertension, any type of regular medication, and cerebrovascular malformations.

### Instrumentation

Subjects received peripheral venous cannulation, ECG, respiration, peripheral oxygen saturation with finger sensor on non-dominant hand (Vital-Guard 450C, Ivy Biomedical Systems, Inc., Branford, CT, United States), cerebral oxygen saturation with near-infrared spectroscopy (NIRS) on the forehead (NIRO cerebral oximeter, Hamamatsu Photonics, K.K., Japan). The right middle cerebral artery (MCA) was insonated through the temporal window with a 2-MHz probe (Delica 9UA with robotic probe, Delica, Shenzen, China) that was maintained at a constant position by a headset. Continuous blood pressure (BP) was measured by volume clamp method on the middle finger of the non-dominant hand and verified using oscillometric determined brachial BP on the contralateral arm (Finapres NOVA, Finapres Medical Systems BV, Enschede, the Netherlands). Stroke volume and cardiac output were provided by the device through non-calibrated pulse-contour analysis. Additionally, we measured stroke volume for cardiac output calculation *via* transjugular ultrasound (Vivid iq, GE Healthcare, Chicago, United States) after 1 minute on every LBNP and LBPP level. For this, the ascending aorta was localized with B-mode ultrasound and three cycles of velocity-time integral measurement were performed with the continuous wave function and the of the device. The left ventricular outflow tract diameter had been measured prior to the experiment. With the mean values of the velocity-time integral and left ventricular outflow tract diameter, stroke volume and cardiac output were calculated (with heart rate from the ECG monitor) for each pressure level.

**FIGURE 1:**
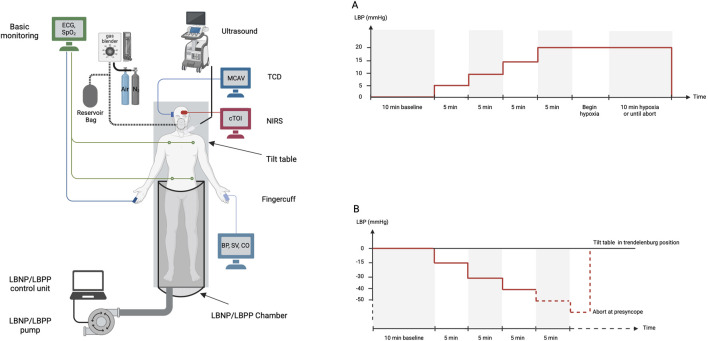
Experimental setup to study response of cerebral blood flow, brain tissue oxygenation, cardiovascular parameters to lower body positive pressure (Protocol **A**) or negative pressure and hypoxia (Protocol **B**). ECG electrocardiogram, SpO_2_ peripheral oxygen saturation, TCD transcranial Doppler, MCAV middle cerebral artery velocity, NIRS near infrared spectroscopy, cTOI tissue oxygenation index by NIRS, BP blood pressure, SV stroke volume, CO cardiac output, LBP lower body pressure, LBNP lower body negative pressure, LBPP lower body positive pressure.

The experimental table was a tilt table with a custom-made lower body pressure chamber. The subject was placed inside the chamber with a neoprene sealing at the level of iliac crest to ensure airtight connection between lower part of the subject and the pressure chamber. Lower body positive and negative pressure was achieved with an air pump connected to the pressure chamber. The air pump was controlled by computer feedback controller using a pressure sensor attached inside the chamber. Experimental setup and protocol are shown in Figure 1. The sequence of the suction protocol was not randomized in favor of performing LBPP and hypoxia prior to the LBNP protocol which induces presyncope.

### LBPP and hypoxia protocol

During the experiment with positive pressure, subjects wore a face mask for in- and expiratory O_2_ and, etCO_2_ measurements which were acquired with an Innocor gas analyzer (Innovision, Glamsbjerg, Denmark).

Once stable signals were achieved on all channels, a baseline measurement of 10 min was started. A LBPP of 5 mmHg was then established for 5 min. LBPP were further increased in 5 mmHg steps every 5 min up to 20 mmHg. The experiment was continued under hypoxia for 10 min or until abort by the subject (Figure 1, panel A). The experiment was terminated by stopping LBPP and detaching the face mask.

Hypoxia was achieved with a gas blender attached to pressurized air and pressurized nitrogen. The subject subsequently inhaled the accumulated mixture gas from a reservoir bag. To achieve optimal hypoxic conditions, an inhaled FiO_2_ of ca. 0.1 was targeted by the experimenter operating the gas blender. Hypoxia was reached with an SpO_2_ of 84%.

#### LBNP protocol

For the LBNP protocol, subjects were placed again on the experimental table as mentioned above. Monitoring was identical to the LBPP experiment.

Once stable signals were present on all channels, recording of baseline measurement was initiated for 10 min. After baseline, lower body pressure was decreased to −15 mmHg for a time of 5 min, and −30 mmHg for another 5 min. LBNP was further decreased in −10 mmHg steps every 5 min until subjects reached presyncope. The onset of presyncope was indicated by the subject experiencing dizziness, nausea, weakness or other signs of presyncope. At presyncope, suction was stopped and the subject was tilted into Trendelenburg position for immediate recovery (Figure 1, panel B).

#### Data acquisition and processing

Hemodynamic data were recorded using the WINDAQ data acquisition system (DI720, DATAQ, Akron, OH, 14 Bit, 500Hz). Sampling time of the NIRO device was set to 0.2 s, internal delay of the NIRO is < 10 m. Data were processed off-line using a custom written software (PV-wave, Visual Numerics Inc., Houston, TX) and Matlab (Mathworks, Natick, MA, United States. Detected beat-to-beat values of R-R intervals (RRI), blood pressure, and transcranial Doppler flow were verified visually. Beat-to-beat values were interpolated and low-pass filtered (cutoff 0.4 Hz) and resampled at 5 Hz for further processing. Cross spectra, coherence and transfer function analysis were used to capture interrelationships between two signals of blood pressure and NIRS or TCD.

#### FFT based transfer function for stationary data

Stationary data segments without any signs of presyncope of 300 s were used for spectral analysis. Linear trends were removed, and power spectral density was estimated with the FFT-based Welch algorithm. The total power (TP) and the power in the very low (VLF: 0.004 to <0.04 Hz), low (LF: 0.04 to <0.15 Hz), and high (HF: 0.15 to <0.40 Hz) frequency ranges were calculated according to TASKFORCE recommendations (Malik, 1996). The LF range was chosen because this is the range where vessels are able to response with neural mediated vasomotion. Blood pressure oscillations above 0.15 Hz are mainly mechanically caused by respiration (Pagani et al., 1986; Diedrich et al., 2003; Brychta et al., 2007).

The stationary transfer function gain was determined as the mean magnitude value of the transfer function in the VLF, LF, and HF bands. Cerebral autoregulation gain (Gxy) between systolic blood pressure and cerebral tissue oxygenation index (cTOI) was estimated (without any criteria limits) and expressed in unit “%/mmHg”.

#### Modified continuous wavelet based transfer function

The Continuous Wavelet Toolkit of Grinsted et al. was modified to allow undistorted power estimation and estimation of transfer function gain (Grinsted et al., 2004). We used simulations with surrogate and real data of TCD and NIRS to test that power and gain were estimated correctly. Details about modified continuous Wavelet based transfer function, simulation with surrogate data, and test with real data are described by Kulapatana et al., 2024 (Kulapatana et al., 2024). Application of this algorithm to TCD and NIRS signals are available in Supplementary Datasheet 1.

#### Comparison between wavelet and FFT based transfer functions

Stationary segments of 5 min during resting supine baseline, lower body negative pressure (LBNP) and lower body positive pressure (LBPP) without presyncopal signs were chosen for estimation of spectral density function, phase, and gain. Wavelet based and FFT based (Welch) algorithm estimates of power, coherence, gain response between signals of blood pressure and cerebral oxygenation were compared. Comparisons were performed using both systolic values and mean values of the signals. We presented analysis using systolic values because it presented the maximal pulsatile component of the blood pressure signal which was the strongest stimulation for cerebral control. Analyses using mean values are provided in Supplementary Datasheet 3.

#### Statistical analysis

For descriptive statistics of and graphical presentation of global hemodynamic and cerebral variables, mean values were calculated across all values recorded during the full duration of each pressure level. Paired t-tests were conducted on hemodynamic and cerebral parameters as part of an exploratory analysis. Agreement between FFT and wavelet based methods were done using repeated-measure Bland-Altman analysis with mixed model for measurements with multiple observations per individual (Bland and Altman, 2007; Parker et al., 2016). Linear correlation analyses was performed using repeated measures correlation which adjust correlation for repeated measurements (Bakdash and Marusich, 2017). P values of <0.05 (two-sided) were considered significant.

## Results

We enrolled five healthy male subjects (age 24.4 ± 2.6 years, body mass index 25.4 ± 1.7 kg/m^2^) who completed the LBPP protocol with additional mild hypoxia followed by a lower body negative pressure LBNP protocol. In all LBNP experiments, presyncope was reached at LBNP levels between −40 and −70 mmHg in all participants. Data of −60 mmHg and −70 mmHg were not further processed because of low number of subjects for each data point. In the LBPP experiments, one subject did not tolerate prolonged exposure to hypoxia, so the experiment had to be terminated early. Although the LBPP experiment was performed before LBNP, the order of presentation of results is reversed below for reasons of better readability.

### Analysis of stationary data

#### Time course of hemodynamic parameters

**FIGURE 2:**
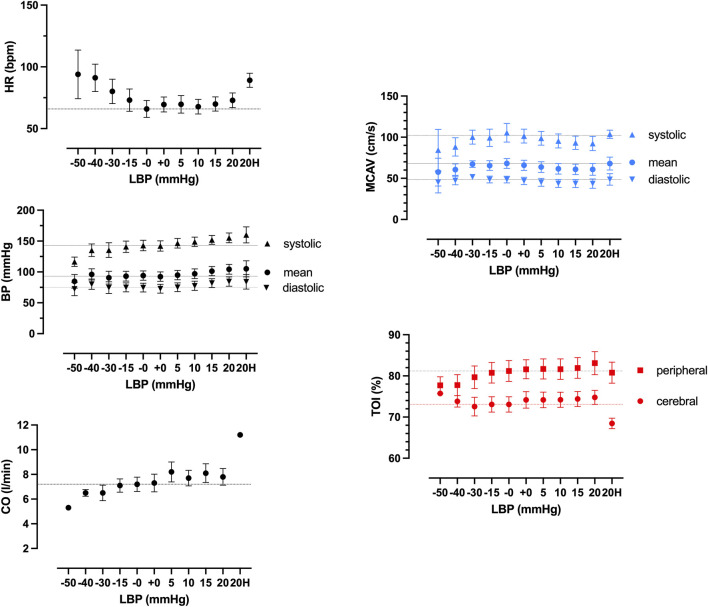
Hemodynamic responses to different pressure levels of negative (LBNP), positive (LBPP) lower body pressure (LBP) and additional hypoxia (20H) of heart rate (HR), blood pressure (BP), cardiac output (CO), middle cerebral artery velocity (MCAV) by transcranial doppler, and cerebral and peripheral tissue oxygenation (TOI) by near infrared spectroscopy. Results show mean values of all subjects.

Averaged hemodynamic response and changes in cerebral blood flow and oxygenation for all LBNP and LBPP pressure levels are shown in Figure 2 and Supplementary Datasheet 3. During decremental pressure protocol (suction), blood pressure and cardiac output decreased, while heart rate increased. During incremental pressure protocol, mean blood pressure (MBP), cardiac output (CO), and heart rate (HR) increased. Mean middle cerebral artery velocity (MCAV_mean_) and cerebral tissue oxygenation index (cTOI) did not change in the pressure range from −40 mmHg to +20 mmHg. There was a marginal decrease of MCAV_mean_ and increase in cTOI during −40 mmHg and −50 mmHg. During pressure level of 20 mmHg and additional hypoxia, MCAV_mean_ decreased while NIRS increased, although these changes did not reach significance. During hypoxia, CO as measured by ultrasound increased significantly (p = 0.006).

#### Global wavelet spectra

**FIGURE 3:**
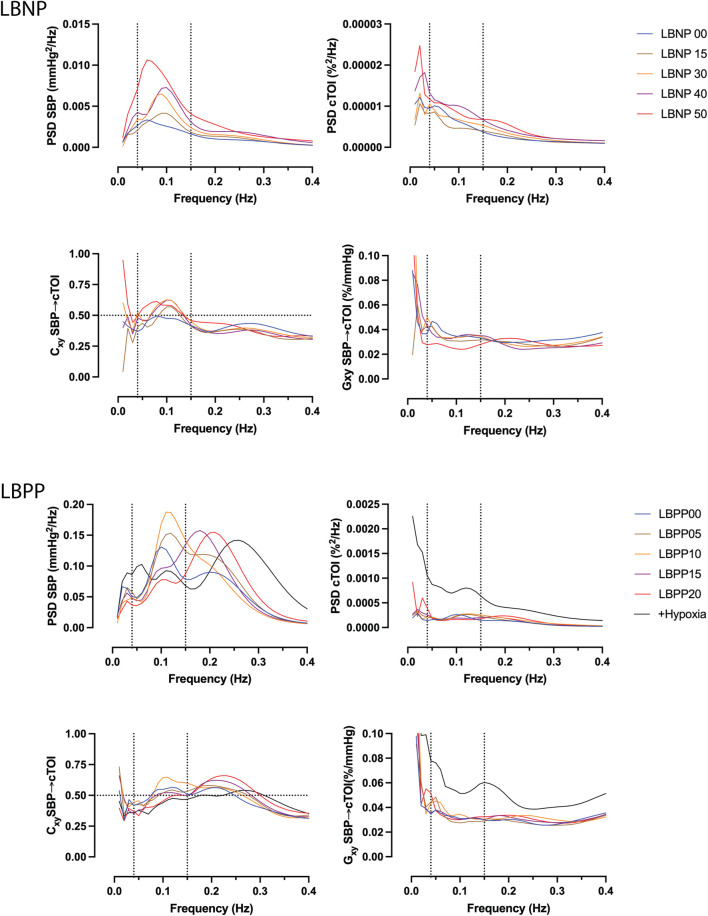
Averaged global wavelet power spectral density (PSD) of systolic blood pressure (SBP, top left) and cerebral tissue oxygenation index (cTOI) by near infrared spectroscopy, magnitude squared coherence (Cxy, bottom left), and transfer function gain (Gxy) during different levels of lower body negative pressure (LBNP, 0 to −50 mmHg), lower body positive pressure (LBPP, 0 to +20 mmHg), as well as additional hypoxia (+Hypoxia).

Averaged global wavelet power spectral density (PSD), coherence, and transfer function gain of blood pressure and NIRS during LBNP and LBPP are shown in Figure 3. During LBNP, PSD increased gradually, especially in the LF frequency range. Coherence increased and reached levels above 0.5 in the LF range. During LBPP, LF peak decreased and high frequency (HF) peak increased. Coherence increased above 0.5 in the LF and HF ranges.

#### Time course of spectral parameters

**FIGURE 4:**
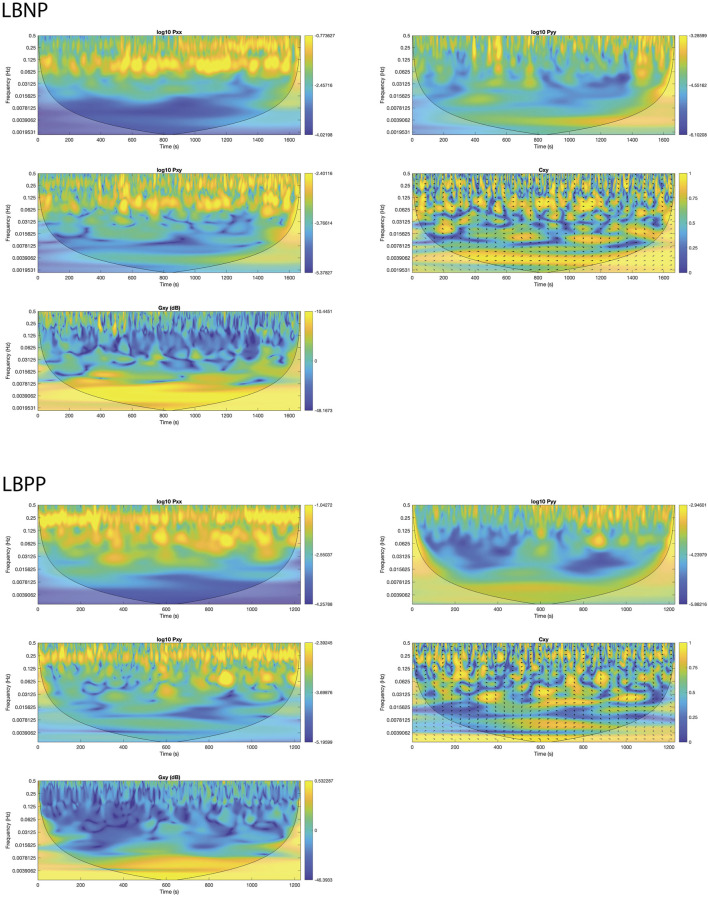
Example of wavelet spectrogram of systolic blood pressure (SBP) and cerebral tissue oxygenation index (cTOI) by near infrared spectroscopy during negative (LBNP) and positive (LBPP) lower body pressure protocols. Images show time courses of SBP autospectra (Pxx), cTOI autospectra, cross spectra (Pxy), coherence (Cxy), and transfer function gain (Gxy) in the different frequency ranges as calculated with the modified continuous wavelet transform algorithm.

A representative wavelet spectrogram showing the time course of autospectra, cross spectrum, coherence, and transfer function gain during the LBNP and LBPP experiment can be seen in Figure 4.

**FIGURE 5:**
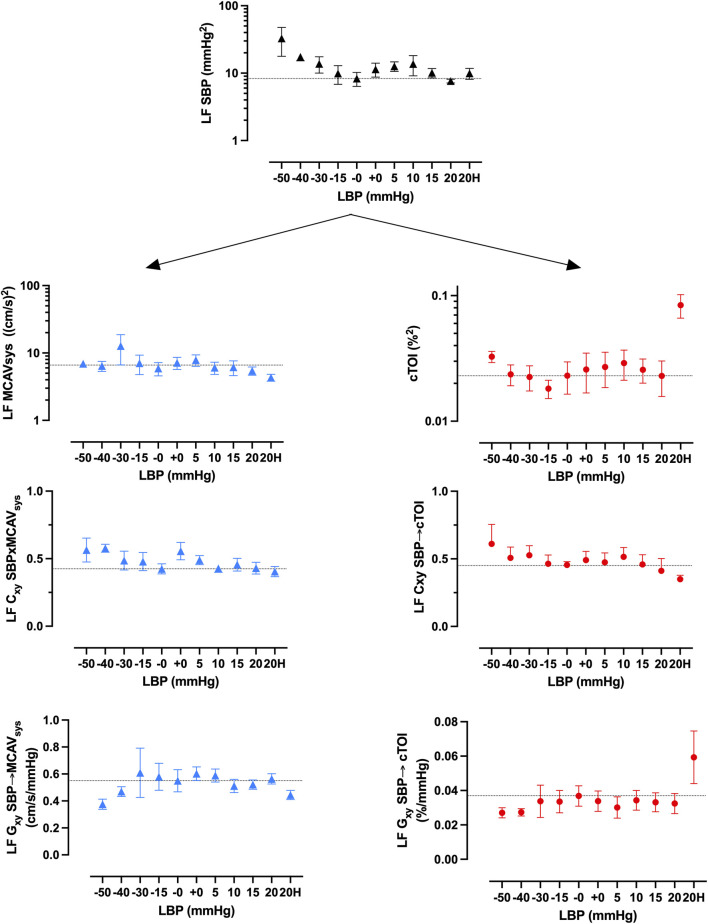
Averaged low frequency (LF) wavelet spectral power of systolic blood pressure (SBP) fluctuations, coherence (Cxy), and transfer function gain (Gxy) between SBP and systolic middle mean cerebral artery velocity (MCAV_sys_) by transcranial Doppler (left, blue) and cerebral tissue oxygenation index (cTOI) by near infrared spectroscopy (right, red).

Figure 5 shows wavelet analysis results of averaged time course of power, coherence, and gain between blood pressure and TCD and blood pressure and NIRS in the LF range. Results of other frequency ranges are presented in the supplement.

LF blood pressure power increases with negative suction. Positive pressure has marginal effects on LF (Figure 5) but increases HF (Supplementary Datasheet 3). LF NIRS spectral power increases during suction while response during pressure is inconclusive. Hypoxia increases LF NIRS spectral power significantly. LF TCD spectral power increases during LBNP and increases during LBPP. LF coherence between blood pressure and NIRS was above 0.5 from −30 mmHg LBNP, increases with further suction and decreases during positive pressure steps. There was a marginal trend of decrease of coherence between blood pressure and TCD. Transfer function gain between blood pressure and NIRS shows a decrease during suction and a significant increase during hypoxia. Transfer function gain between blood pressure and TCD show marginal decreases during suction and positive pressure.

#### Comparison of wavelet and welch results

**FIGURE 6:**
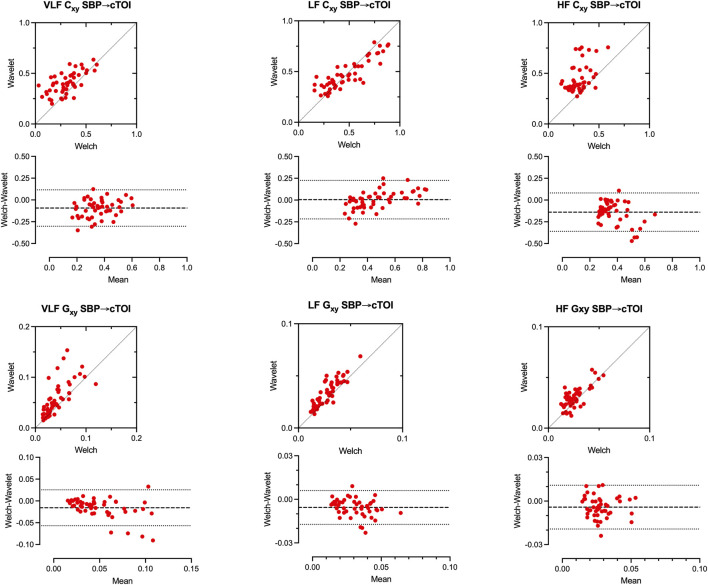
Comparison of Wavelet based and FFT based estimates (Welch) of squared magnitude coherence (Cxy) and transfer function gain (Gxy) between systolic blood pressure (SBP) and cerebral tissue oxygenation index (cTOI) by near infrared spectroscopy (NIRS) in the very low frequency (VLF, left), low frequency (LF, middle), and high frequency range (HF, right) during negative and positive lower body pressure. Broken lines represent confidence interval ±2SD in correlation and Bland-Altman plots. Results show data of all subjects.

A comparison between the wavelet and FFT based estimates (Welch) of squared magnitude coherence (Cxy) and transfer function gain (Gxy) between blood pressure and NIRS indicates good agreement in Bland Altman Plots for repeated measurement in the LF range (bias [95% confidence interval], Cxy: 0.004 [-0.217, 0.225], Gxy: 0.006 [-0.017.0.006]). The Bland Altman agreements were less in VLF range (Cxy: 0.093 [-0.302.0.115], Gxy: 0.016 [-0.057.0.025]) and HF range (Cxy: 0.139 [-0.360.0.081], Gxy: 0.004 [-0.019.0.011]). Linear correlations were present, especially in the LF range (Cxy: r = 0.69, p < 0.001, Gxy: r = 0.74, p = 0.001), but less in the VLF range (Cxy: r = 0.65, p < 0.001, Gxy: r = 0.66, p < 0.001) and HF range (Cxy: r = 0.39, p < 0.001, Gxy: r = 0.71, p = 0.001). FFT based coherence estimates were smaller than wavelet estimates for values <0.5 (Figure 6).

Similar agreements could also be found for the TCD signal as shown in Supplementary Datasheet 4.

#### Comparison of LF power, coherence and gain in TCD and NIRS signal

**FIGURE 7:**
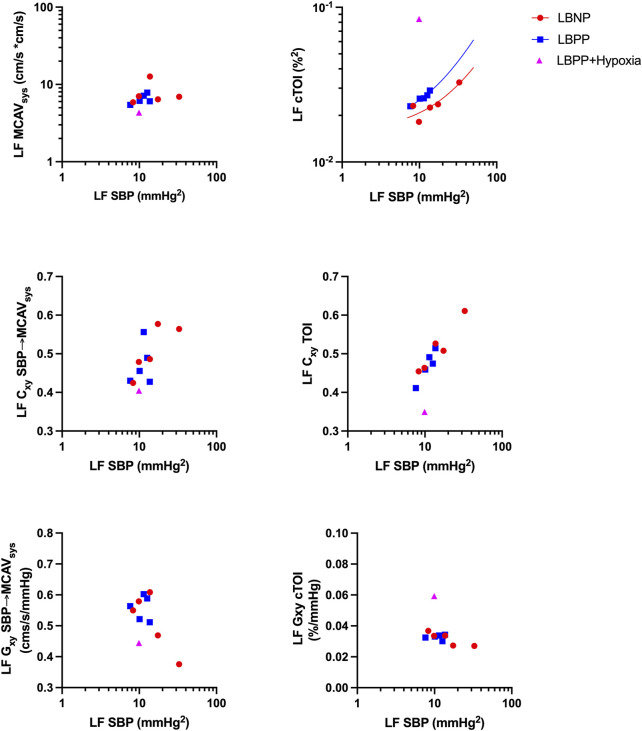
Relationship between low frequency (LF) fluctuations in systolic blood pressure (SBP) and LF spectral power, coherence (Cxy), and transfer function gain (Gxy) of systolic mean middle cerebral artery velocity (MCAV_sys_) and cerebral tissue oxygenation (cTOI) during lower body negative pressure (LBNP), lower body positive pressure (LBPP) and additional hypoxia. Results show mean values of all subjects on the respective pressure level or hypoxia.

There exists a positive correlation of LF oscillation and between systolic blood pressure and cTOI. Positive pressure produces higher LF oscillation in cerebral TOI at the same level of LF SBP (Figure 7, upper right). This is less prominent in cerebral blood flow velocity (Figure 7, upper left). Higher LF SBP is in concordance with higher LF coherence in cTOI.

**FIGURE 8:**
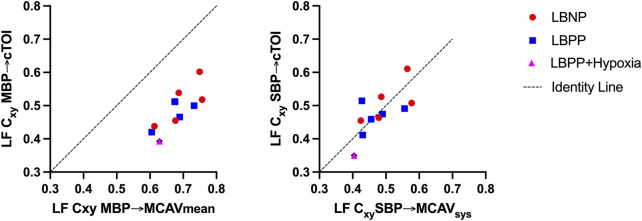
Comparison of low frequency (LF) coherence (Cxy) between mean blood pressure (MBP) and mean middle cerebral artery velocity (MCAV_mean_) and cerebral tissue oxygenation index (cTOI) (left) and between systolic blood pressure (SBP) and systolic middle cerebral artery velocity (MCAV_sys_) and cerebral tissue oxygenation index (cTOI). Results show mean values of all subjects on the respective pressure level or hypoxia.

Results of LF Coherence between TCD and NIRS-derived continuous wavelet transform showed good agreement between TCD and NIRS However, this applied when using systolic blood pressure and peak systolic flow velocity instead of mean blood pressure or mean velocity as parameters as shown in Figure 8.

## Discussion

With the advent of NIRS as a surrogate parameter for cerebral blood flow, several NIRS-based cerebral autoregulation measurements have been described. Some time domain analyses use a moving correlation coefficient to describe the linear relationship between NIRS and MBP. The Mx or COx calculation based on a moving Pearson correlation coefficient has been used on many occasions, even in clinical practice (Soehle et al., 2004; Brady et al., 2010). With these methods, changes can be observed in a certain frequency range, but more rapid or slower changes remain unnoticed. Other frequency domain methods describe the phase relationship, gain and the transfer function between MBP as an input and NIRS as an output signal. These methods can be used to describe autoregulation properties in a wide range of frequencies. However, temporal resolution remains limited.

Only recently, the continuous wavelet transform has been introduced to evaluate the relationship between MBP and NIRS. The use of the continuous wavelet transform provides some advantages. One is an optimal time-frequency resolution, providing the opportunity to see results both in the time and frequency domain. The other is the wide range of frequency bands that can be analyzed simultaneously. However, there are obstacles in the implementation of such a method. Wavelets require a continuous signal and missing data points have to be interpolated. Additionally, implementation of the method requires extensive background knowledge when compared to the much simpler methods based on moving correlation indices. The Matlab CWT function does not include corrections of power distortion, nor does it calculate gain. Since the source code is not available for the user this cannot be easily corrected. Tian et al. presented a cohort of critically ill neonates with NIRS-derived autoregulation assessment based continuous wavelet transform. The authors focus on significant coherence between blood pressure and the NIRS signal as a representation of impaired autoregulation. A decrease in phase shift and increase in transfer function gain is also described as a correlate of autoregulation impairment (Tian et al., 2016). The calculations of Tian et al. have been performed based on a tool originally presented by Grinsted et al. (Grinsted et al., 2004) This tool, originally intended for geophysical time series analysis is designed to focus on wavelet coherence. To isolate regions of significant coherence, Grinsted’s function compares the signals to signal consisting of red noise. Since the source code is freely available, we also used Grinsted’s algorithm for the analysis of our subjects. However, for autoregulation analysis, substantial modifications were necessary to not only obtain coherence, but also unbiased power estimates and transfer function gain. A comparison to the common FFT based transfer function results show the validity of the wavelet-based method as can be seen in Figure 6. Tian’s method focuses exclusively on the very low frequency range, observing changes over hours which makes their method difficult to compare to the one presented here. Another CWT-based method of autoregulation assessment has been introduced by Liu et al., producing a NIRS derived wavelet-based autoregulation index, or wCOx. While this method has been validated in piglets in comparison to correlation based methods and provides a simple measure of autoregulation, wCOx also works exclusively in the VLF range (Liu et al., 2020). Both the methods presented by Tian et al. and Liu et al., as well as a method presented by Rajaram et al. focus on coherence which is the linear relationship between two signals, thus reflecting one component of cerebral autoregulation (Tian et al., 2016; Liu et al., 2020; Rajaram et al., 2022). Another component which is rarely reported is the amplification or damping of the input signal (blood pressure) to output signals (cerebral blood flow surrogates) as represented by transfer function gain.

We demonstrated good agreement in power, coherence, transfer function gain estimates between FFT based Welch method and our modified wavelet method. This was confirmed in simulated data and in stationary real data in healthy males undergoing LBNP until presyncope and LBPP with added hypoxia on the highest pressure level. Transfer function analysis using continuous NIRS-derived wavelet transform could be useful for exploring cerebral autoregulation dynamics, especially where stationarity is not present. We found higher wavelet coherence values in real data especially in the VLF and LF range which might be caused by non-stationarity and underestimation of parameters by the FFT based Welch method. We also found that using systolic blood pressure signal will improve the agreement between Welch and Wavelet coherence estimates. In addition, TCD and NIRS-derived coherence showed good agreement only when using TCD peak flow velocity and systolic blood pressure instead of mean flow and mean blood pressure (Figure 8). Cxy SBP-MCAV as well as Cxy SPB-cTOI showed good agreement in Bland Altman Plot and good linear agreements with changes in SBP as shown in Figure 7. The above findings suggest that SBP and systolic flow velocity in TCD may be more useful in determining cerebral autoregulation integrity, at least in the LF range, for the method described here. This is in contrast to most previous reports relying on MBP in conjunction with NIRS or mean MCAV (Tian et al., 2016; Zhang et al., 1998a). So far, only poor agreement could been shown between TCD and NIRS-derived transfer function calculations (Panerai et al., 2023). However, these results were exclusively focused on phase shift as a parameter of cerebral autoregulation (Mol et al., 2021). While our results were focused more on coherence and gain, we could show improved of agreement between coherence values by comparing systolic values of MCAV and SBP, which has not been reported previously and which may have implications for further studies. One could also suspect improved agreement between TCD and NIRS by using the CWT due to the optimized time-frequency resolution which is especially important in non-stationary data. However, this remains to be confirmed.

Furthermore, our results suggest that most changes of coherence and transfer function gain during the lower body pressure protocol occurred in the low frequency range. High frequency oscillations are increasing during LBPP but had no effect on gain. This is plausible since smooth muscle of the vessels have an operational low pass cutoff at ca. 0.1 Hz. Recommendations for assessment of cerebral autoregulation suggest a frequency range beyond 0.1 Hz. This may have consequences because the signals are contaminated by mechanically induced respiratory oscillations. Our results are somewhat in contrast to previous reports. In previous reports, increased coherence together with increased gain and decreased phase shift were interpreted as reduced cerebrovascular responsiveness (Zhang et al., 1998b). While we can confirm this for phase shift and coherence, we found the opposite for transfer function gain. Decreased gain with negative phase shift suggests counter-regulation mechanisms which are more powerful than the pressure-passive behavior of cerebral perfusion close to presyncope. However, it has to be mentioned that most previous studies used transcranial Doppler sonography as a surrogate for cerebral blood flow which has been shown to be more volatile than NIRS. Our results also suggest that the LF range is of the most importance for assessment of cerebral autoregulation since both analyses based on blood pressure fluctuations as well as on lower body pressure levels show the most distinct increases in coherence. With LBPP, we could not observe an impairment in cerebral autoregulation parameters. On the contrary, coherence analysis saw a slight decrease with increasing pressure levels. Perry et al. showed increased cerebral blood velocity with 20 mmHg, but not with 40 mmHg of LBPP, but does not provide a measurement of cerebral autoregulation (Perry et al., 2013). In our cohort of healthy male adults, increased cardiac preload does not appear sufficient to reach nor to exceed the upper autoregulation limit.

During Hypoxia, which was added to LBPP, we observed an increase in LF transfer function gain without an increase in coherence or decrease in phase shift. We interpret this isolated increase as a local vasomotion phenomenon rather than impaired cerebral autoregulation. Ainslie et al. reported impaired autoregulation which was indicated by decreased LF phase or increased LF gain between arterial pressure and transcranial Doppler-derived middle cerebral artery flow velocity (Ainslie et al., 2008; Ainslie et al., 2007). Similar results have been presented by Subudhi et al. (Subudhi et al., 2009) Transfer function analysis might be limited during hypoxia because of the nature how the NIRS signal is generated. We found extreme values during hypoxia which could be false estimates. This has to be tested in further protocols.

There are certain limitations associated with the results presented here. This is a validation study with a small sample size. Results should be interpreted carefully. Since only young adult male participants were included, we are unable to transfer results to other age groups. While we deliberately recruited only male subjects, sex differences in cerebral autoregulation may play an important role and results cannot be generalized at this point. This will have to be addressed in a future study. It also has to be noted that all methods used in this study to represent cerebral blood flow are surrogate parameters. The application of NIRS in this context has long been subject to debate. This is due to many factors influencing the NIRS signal and its susceptibility to extracranial contamination which may influence measurements to a certain extent and which cannot be addressed easily. While a large degree of extracranial contamination appears unlikely due to the agreement with TCD measurements, this cannot be ruled out. Another limitation is the use of hypoxia which influences systemic oxygen saturation and may cause cerebral vasodilation and cause underestimation of MCAV. Therefore, results during hypoxia should be interpreted in this context.

In summary, we could show good agreement in power, coherence, and transfer function gain estimates between FFT based method and our modified wavelet method. This could be confirmed in simulated data and healthy subjects undergoing lower body negative and positive pressure with added mild hypoxia. Therefore, our results suggest that NIRS-derived wavelet transform may be useful to assess cerebral autoregulation dynamics, especially in non-stationary data.

## Data Availability

The raw data supporting the conclusions of this article will be made available by the authors, without undue reservation.
