# Supplementary material for: Continuous wavelet based transfer function analysis of cerebral autoregulation dynamics for neuromonitoring using near-infrared spectroscopy
Source: Front Physiol. 2025 Jun 18;16:1616125. doi: 10.3389/fphys.2025.1616125 (PMC12213380; doi:10.3389/fphys.2025.1616125)
Supplement: Supplementary file 1 [file DataSheet2.pdf]

Validation of Wavelet Transfer Function with Simulation of Stationary Surrogate Signals

André Diedrich and Surat Kulapatana, Vanderbilt University, Nashville, TN, USA

Background

Wavelet based methods have been introduced for multivariate spectral analysis. Grinsted et. al developed software for coherence and phase analysis of the time series data of river elevations (Grinsted, Moore, and Jevrejeva 2004). The open-source code has been translated in several software languages and been applied for transcranial doppler signal (TCD), near infrared spectroscopy (NIRS), and other cardiovascular signals.

The limitation of current open-source Matlab software package (http://grinsted.github.io/wavelet-coherence/) from Grinsted are:

a) no implementation of power and transfer function output,
b) wavelet power calculations were biased (Liu, San Liang, and Weisberg 2007; Veleda, Montagne, and Araujo 2012), and
c) no validation in physiological frequency ranges for cardiovascular signals (LF: 0.04-0.15 Hz, HF: 0.15-0.40Hz).

We extended the open-source Matlab code of Grinsted with output of estimated transfer function gain. We rectified the bias in the wavelet power spectrum for auto-spectra power, and cross-spectra power as proposed by Liu (https://ocgweb.marine.usf.edu/~liu/wavelet.html). Details are available in supplement "Wavelet Transfer Function Estimation with Modified Cross Wavelet Function from Grinsted".

The purpose of this paper is to validate the modified code with the requirement that power, gain, phase, coherence estimates are equal to estimates with conventional FFT-based transfer function method for surrogate stationary signals.

Methods

We generated two surrogate stationary signals (x, y) as combination of two sinusoidal components at two frequencies f1 (f1 = 0.1 Hz) and f2 (f2 = 0.2Hz) in physiological ranges of cardiovascular parameters. These frequencies are selected to simulate sympathetic induced modulation of blood pressure with 10 seconds periodicity in the low frequency range (LF: 0.04-0.15Hz, Mayer waves) and mechanical induced respiratory fluctuations of blood pressure in the high frequency range (HF: 0.15-0.4 Hz) in humans (Diedrich et al. 2003; Brychta et al. 2007). Signal x represents surrogate of blood pressure. Signal y represents surrogate of cerebral blood flow.

The relative local phase (Axy) between two signals components was set $-\pi/2$ for 0.1 Hz and $+\pi/2$ for 0.25Hz. A negative phase means that that x leads y. A positive phase means that y is leading relative to x. The local gain (Gxy) between two signal components was set 0.5 (amplitude ratio:

a11/a21 = 0.5) at frequency f1 and 1 (amplitude ratio: a12/a22 = 1) at frequency component f2 (formula [1]).

```
x(t) =   a11 * sin(2π*f1*t) + a12 * sin(2π*f2*t);                        [1]
y(t) =   a21 * sin(2π*f1*t+phi1) + a22 * sin(2π*f2*t+phi2), where
f1 = 0.1Hz, phi1 = −π/2
f2 = 0.25Hz, phi2 = +π/2
Gxy1 = a11/a21 = 0.5, Gxy2 = a21/a22 = 1
```

We added white noise with 0.1 variance of both surrogate signals. We calculated the true partial power of each sinusoidal component using equation [1] and true total power using variance of surrogate signals averaged over 100 simulations.

Results

Figure 1 shows the local wavelet spectrogram of the simulated input (x) and output signal (y) with visible components at 0.1Hz and 0.25 Hz. The phases are represented as small arrows. If the arrow points down, then the phase is negative (x leads y). If the arrow points up, then the phase is positive (x lags y). Horizontal right arrow represents in-phase (zero-phase). Left arrow represents anti-phase. The coherence plot shows that the phase is positive at the HF range (arrow up) and negative in the LF range which agrees with our simulated signals.

Figure 2 shows the global spectra, squared coherence, and transfer function gain of both wavelet and FFT based Welch methods. The auto-spectra show clearly peaks at 0.1 and 0.25 Hz. The global phase plot (Axy) shows a negative phase at 0.1 Hz and positive phase at 0.25Hz. The estimated gain was 0.5 at 0.1 Hz and 1 at 0.25Hz. High coherence values at both frequencies indicate a linear relationship.

Table 1 summarizes the averaged results over 100 repeated simulations. There was an excellent agreement between Wavelet and FFT Welch method. All conventional power calculation and estimates of coherence, phase, and gain were not different at both frequencies.

Summary

The modified wavelet-based algorithm showed similar estimates for transfer function gain, spectral power, phase, and coherence for simulated stationary signals in cardiovascular frequency ranges as compared with FFT-based Welch method.

References

Diedrich A, Kulapatan S, "Wavelet Transfer Function Estimation with Modified Cross Wavelet Function from Grinsted". Supplement 1.

Brychta, Robert J., Richard Shiavi, David Robertson, Italo Biaggioni, and André Diedrich. 2007. "A Simplified Two-Component Model of Blood Pressure Fluctuation." *American Journal*

of Physiology-Heart and Circulatory Physiology 292 (2): H1193–1203. https://doi.org/10.1152/ajpheart.00645.2006.

Diedrich, A., J. Jordan, J. Tank, J. R. Shannon, R. Robertson, F. C. Luft, D. Robertson, and I. Biaggioni. 2003. “The Sympathetic Nervous System in Hypertension: Assessment by Blood Pressure Variability and Ganglionic Blockade.” *J Hypertens* 21 (September):1677–86. https://doi.org/10.1097/01.hjh.0000084711.87421.07.

Grinsted, A., J. C. Moore, and S. Jevrejeva. 2004. “Application of the Cross Wavelet Transform and Wavelet Coherence to Geophysical Time Series.” *Nonlinear Processes in Geophysics* 11 (5/6): 561–66. https://doi.org/10.5194/npg-11-561-2004.

Liu, Yonggang, X. San Liang, and Robert H. Weisberg. 2007. “Rectification of the Bias in the Wavelet Power Spectrum.” *Journal of Atmospheric and Oceanic Technology* 24 (12): 2093–2102. https://doi.org/10.1175/2007JTECHO511.1.

Veleda, Doris, Raul Montagne, and Moacyr Araujo. 2012. “Cross-Wavelet Bias Corrected by Normalizing Scales.” *Journal of Atmospheric and Oceanic Technology* 29 (9): 1401–8. https://doi.org/10.1175/JTECH-D-11-00140.1.

Table 1. Comparison of Power, Gain, Phase, and Squared Coherence Estimates using FFT based Welch Method, Wavelet Method, and Variance. Mean and Standard Deviation of n=100 simulations and noise factor 0.1.

	Welch			Wavelet			Variance		
Pxx LF	2.001	±	0.023	1.924	±	0.018	2.001	±	0.000
Pxx HF	0.505	±	0.012	0.539	±	0.010	0.500	±	0.000
Pyy LF	0.501	±	0.004	0.481	±	0.004	0.500	±	0.000
Pyy HF	0.501	±	0.005	0.513	±	0.004	0.500	±	0.000
Pxx Tot	2.562	±	0.026	2.550	±	0.022	2.563	±	0.022
Pyy Tot	1.011	±	0.007	1.014	±	0.005	1.011	±	0.005
Gxy 0.10 Hz	0.500	±	0.004	0.501	±	0.003			
Gxy 0.25 Hz	1.001	±	0.013	1.000	±	0.011			
Axy 0.10 Hz	-1.571	±	0.007	-1.570	±	0.005			
Axy 0.25 Hz	1.570	±	0.012	1.571	±	0.011			
Cxy 0.10 Hz	0.999	±	0.000	0.998	±	0.000			
Cxy 0.25 Hz	0.999	±	0.000	0.994	±	0.001			

Pxx Power of input signal for low (LF: 0.04 - 0.15), high (HF:0.15 -0.4 Hz) frequency range and total power. (Tot: 0-0.4Hz). Pyy Power of signal y for low (LF), high (HF) frequency and total (Tot). Gxy Gain, Axy Phase, and Squared Coherence (Cxy) between x and y at 0.10 Hz and 0.25 Hz

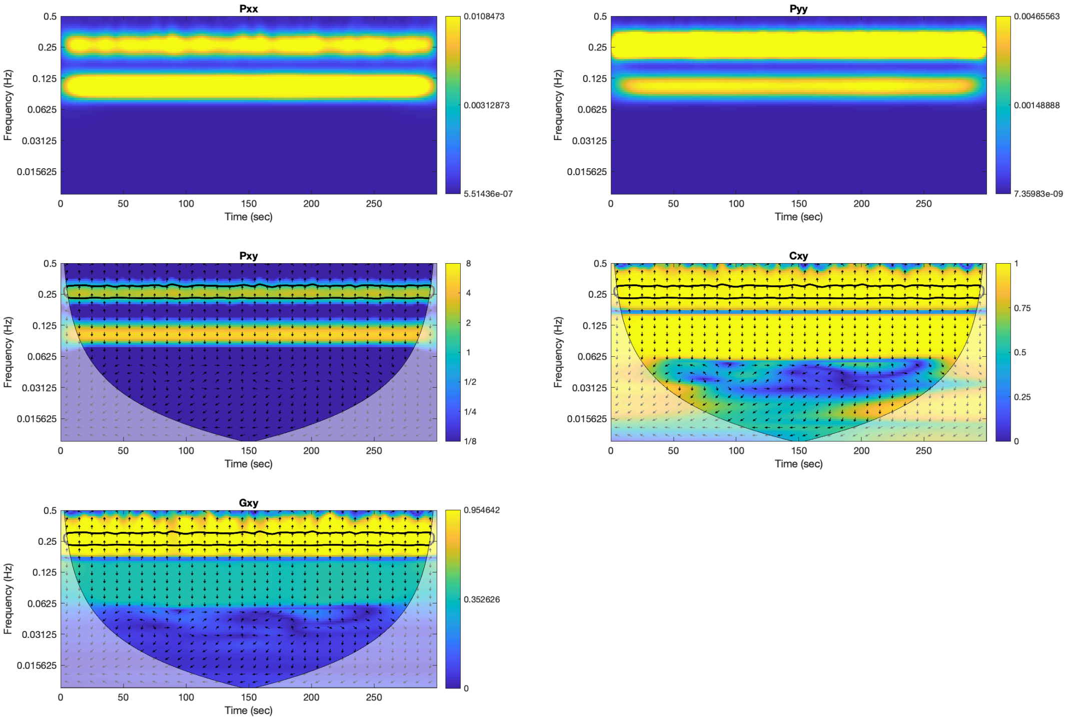

Figure 1. Example output of modified Cross Wavelet Function (xwt_ext) to estimate Transfer Function Gain between simulated sinusoidal input x and output signal y containing two frequencies at components at 0.1 Hz and 0.25 Hz. The local phase (Axy) is represented as a small arrow. Horizontal pointing right arrow represents in-phase (Axy = 0, zero-phase). Horizontal pointing left arrow represents anti-phase (Axy = $-\pi$). Pointing down arrow indicates leading phase with x is leading y (Axy < 0). Pointing up arrow indicates means x is lagging y (Axy > 0). The black contoured area represents significant coherence values different from noise. Axy local phase between x and y, Pxx, Pyy local wavelet auto-spectra; Pxy local wavelet cross-spectra; Cxy squared coherence; Gxy local transfer function gain.

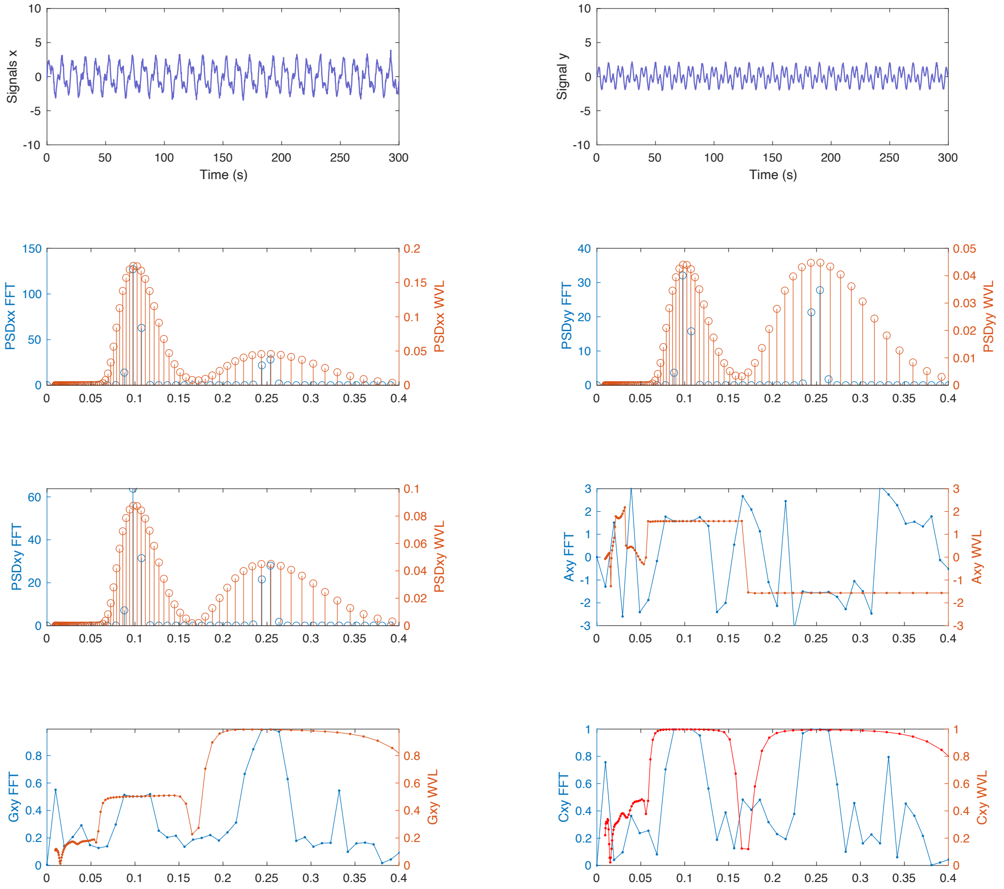

Figure 2. Example of simulated signal x and signal y consist of two frequencies at 0.1 Hz and 0.25 Hz, estimated power spectral density (PSD, first row), cross spectral density (PSDxy) and phase (Axy), gain (Gxy), and squared coherence (Cxy) using FFT based Welch method (FFT, blue) and Wavelet (WVL, red). The input signal was constructed to have power of 2 and 0.5 at 0.10 Hz and 0.25 Hz, respectively. The simulated output signal was constructed to have power of 0.5 at 0.10 Hz and 0.25 Hz. The simulated relative phase was $\pi/2$ and $-\pi/2$ at 0.1 and 0.25 Hz, respectively. The gain was 0.5 and 1 at 0.1 and 0.25Hz. The plots show a good agreement of the estimates of both methods and frequencies (see details in Table1).
