# Supplementary material for: Continuous wavelet based transfer function analysis of cerebral autoregulation dynamics for neuromonitoring using near-infrared spectroscopy
Source: Front Physiol. 2025 Jun 18;16:1616125. doi: 10.3389/fphys.2025.1616125 (PMC12213380; doi:10.3389/fphys.2025.1616125)
Supplement: Supplementary file 2 [file DataSheet4.pdf]

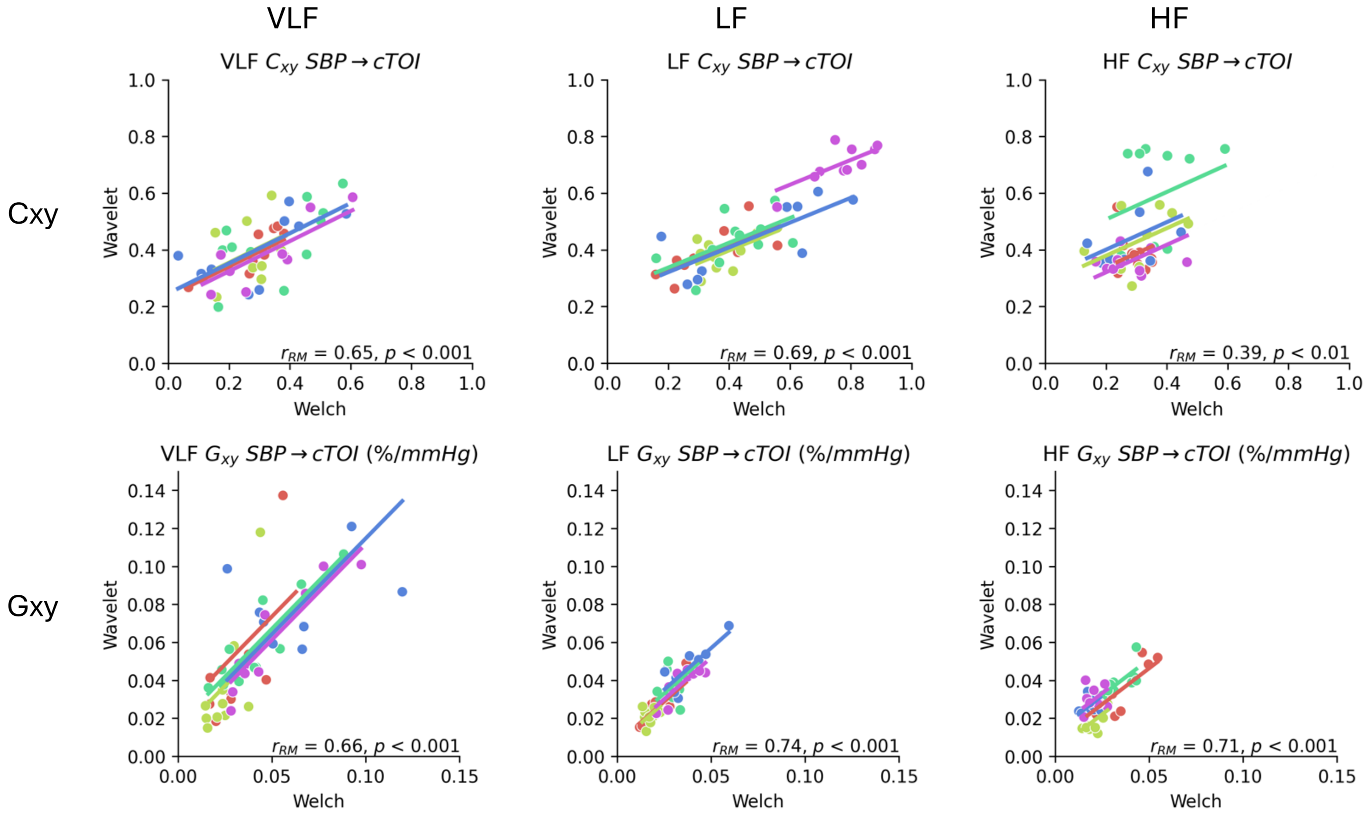

Figure S3. Repeated measurements correlations between FFT-based Welch and wavelet-based estimation of coherence (C_{xy}) and transfer function gain (G_{xy}) of systolic blood pressure and near infrared spectroscopy (NIRS) in the very low frequency, low frequency (LF), and high frequency (HF) range during LBNP and LBPP. Correlation (r_{RM})and p value is adjusted for repeated measures.

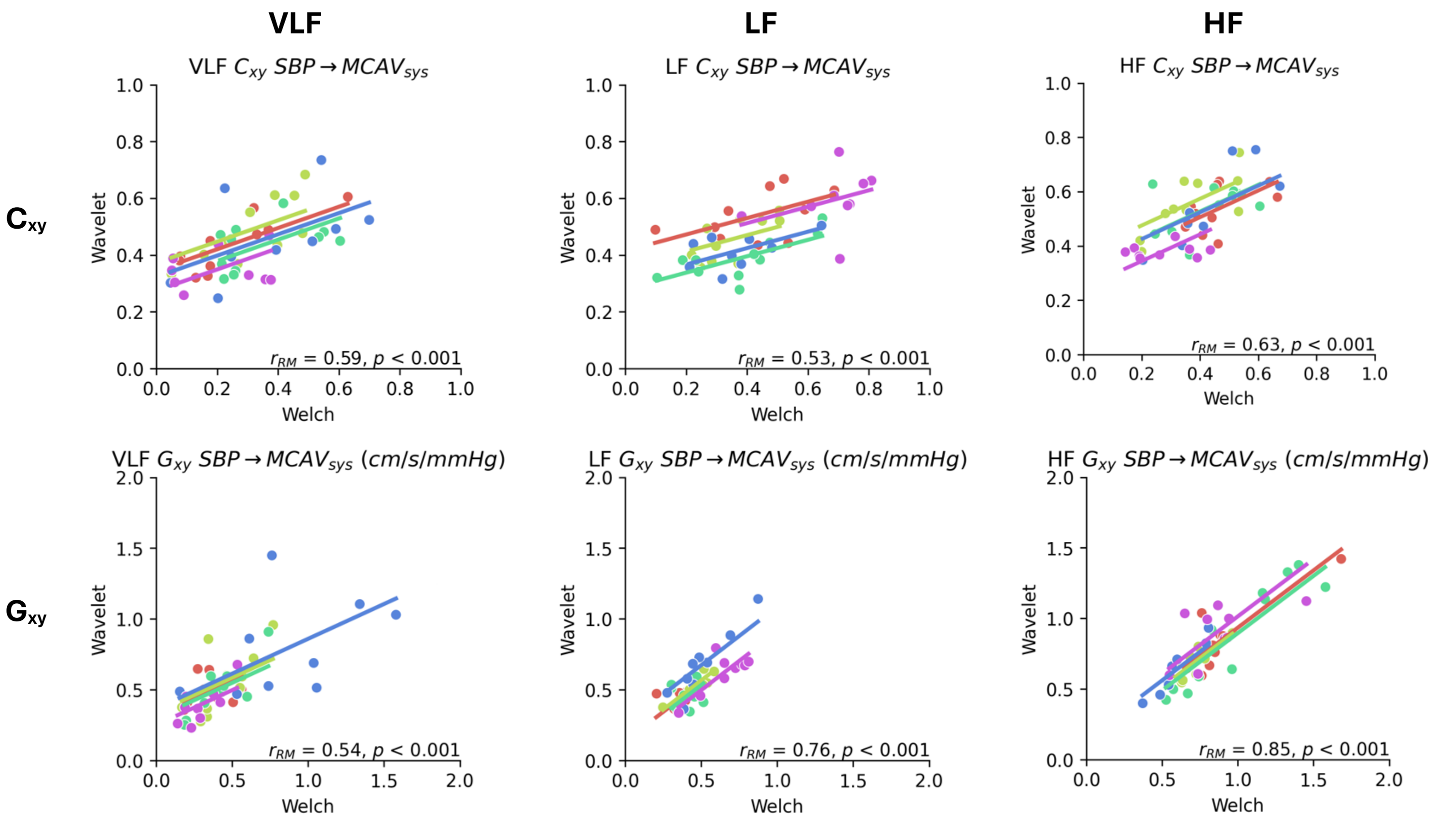

Figure S4. Repeated measures of correlations between FFT-based Welch and wavelet-based estimation of coherence (C_{xy}) and transfer function gain (G_{xy}) of systolic blood pressure and median cerebral artery velocity (MCAV) in the very low frequency, low frequency (LF), and high frequency (HF) range during LBNP and LBPP. Correlation (r_{RM})and p value is adjusted for repeated measures.

Reference:

Bakdash, Jonathan Z., and Laura R. Marusich. 2017. “Repeated Measures Correlation.” *Frontiers in Psychology* 8 (April):456. https://doi.org/10.3389/fpsyg.2017.00456.
