# Supplementary material for: Continuous wavelet based transfer function analysis of cerebral autoregulation dynamics for neuromonitoring using near-infrared spectroscopy
Source: Front Physiol. 2025 Jun 18;16:1616125. doi: 10.3389/fphys.2025.1616125 (PMC12213380; doi:10.3389/fphys.2025.1616125)
Supplement: Supplementary file 3 [file DataSheet3.pdf]

Table S1. Hemodynamic response to lower body negative (LBNP) and positive (LBPP) and additional hypoxia in 5 healthy subjects.

**LBNP**	Baseline (n=5)	-15 mmHg (n=5)	-30 mmHg (n=5)	-40 mmHg (n=5)	-50 mmHg (n=4)		p
HR (bpm)	66 ± 15	73 ± 20	80 ± 22	91 ± 22	94 ± 27		0.167
MBP_{NOVA} (mmHg)	94 ± 17	93 ± 18	91 ± 21	96 ± 18	91 ± 8		0.763
SV_{NOVA} (ml)	90 ± 11	80 ± 9	70 ± 8	62 ± 7	57 ± 5		**0.012**
CO_{NOVA} (l/min)	5.9 ± 1.1	5.8 ± 1.2	5.5 ± 1.0	5.5 ± 0.9	5.4 ± 0.7		0.499
SV_{ECHO} (ml)	102 ± 16	94 ± 13	77 ± 21	68 ± 11	58 ± 11		**0.020**
CO_{ECHO} (l/min)	7.2 ± 1.3	7.1 ± 1.2	6.5 ± 1.4	6.5 ± 0.6	5.3 ± 0.1		0.062
$MCAV_{mean}$ (cm/s)	68.1 ± 13	65.5 ± 13	67.3 ± 8	60.5 ± 14	57.6 ± 24		0.498
cTOI (%)	73 ± 4	73 ± 4	73 ± 4	74 ± 3	76 ± 0.6		0.235

**LBPP**	Baseline (n=5)	5 mmHg (n=5)	10 mmHg (n=5)	15 mmHg (n=5)	20 mmHg (n=5)	20+hypoxia (n=5)	p
HR (bpm)	69 ± 13	69 ± 16	67 ± 13	69± 13	73 ± 13	83 ± 17	0.217
MBP_{NOVA} (mmHg)	92 ± 17	95 ± 17	97 ± 18	101 ± 17	104 ± 18	105 ± 26	0.416
SV_{NOVA} (ml)	95 ± 14	92 ± 13	90 ± 14	85 ± 13	84 ± 14	86 ± 17	0.413
CO_{NOVA} (l/min)	6.6 ± 1.3	6.4 ± 1.5	6.0 ± 1.2	5.9 ± 1.1	6.1 ± 1.3	7.6 ± 1.5	0.323
SV_{ECHO} (ml)	99 ± 22	108 ± 16	104 ± 20	107 ± 17	103 ± 16	122 ± 4	0.083
CO_{ECHO} (l/min)	7.3 ± 1.6	8.2 ± 1.8	7.7 ± 1.4	8.1 ± 1.7	7.8 ± 1.5	11.2 ± 0.0	**0.006**
$MCAV_{mean}$(cm/s)	65.9± 14	63.7 ± 14	61.5 ± 14	61.0 ± 14	60.8 ± 16	68.0 ± 16	0.836
cTOI (%)	74.2 ± 5	74.2 ± 4	74.2 ± 4	74.4 ± 4	74.8 ± 4	68.5 ± 3	0.094

HR heart rate; MBP mean blood pressure; SV stroke volume; CO cardiac output; NOVA by finger cuff volume-clamp device; ECHO by transjugular ultrasound; cTOI cerebral tissue oxygenation index by near infrared spectroscopy. Results are presented as mean ± standard deviation. P-Values present significance level of differences between last LBNP pressure level and baseline, and for differences between LBPP 20 mmHg with additional hypoxia vs. baseline.

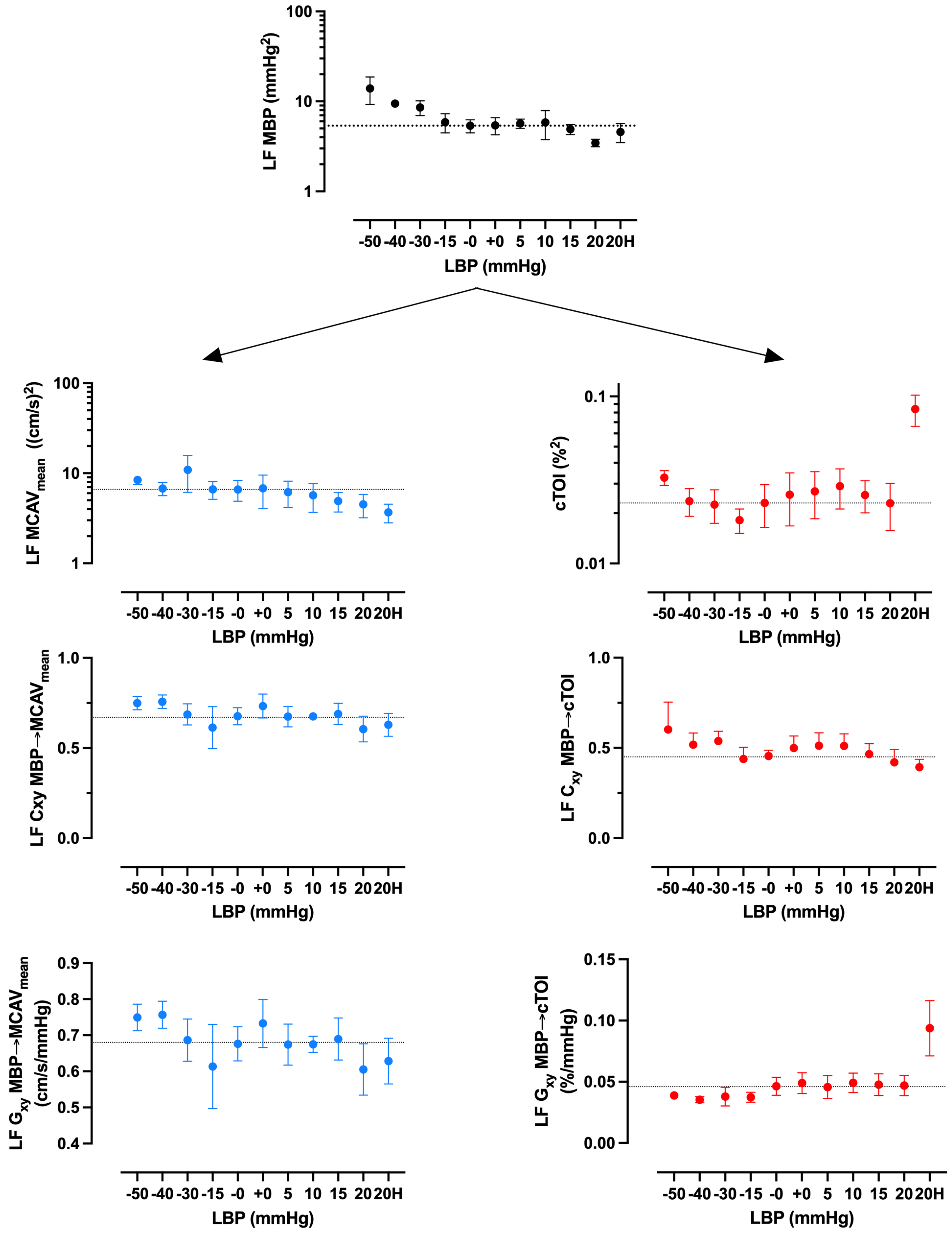

Figure S1. Averaged low frequency (LF) spectral power of mean blood pressure fluctuations (MBP) during different levels of lower body pressure (LBP). Average response in low frequency power, coherence (Cxy, middle row), and transfer function gain (Gxy bottom row) of mean middle cerebral artery velocity (MCAV) by transcranial doppler (left, red) and cerebral tissue oxygenation index (cTOI) by near infrared spectroscopy (right, blue).

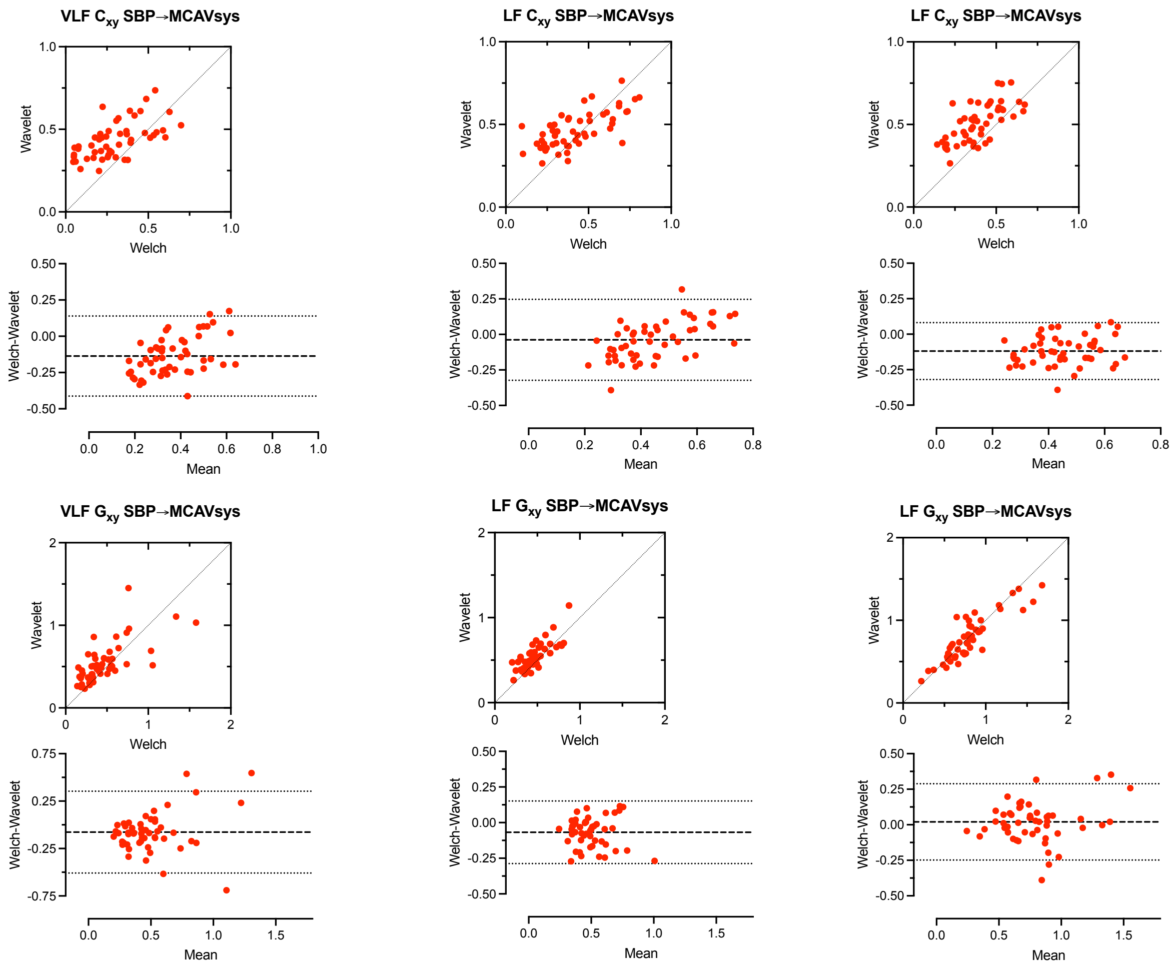

Figure S2. Comparison of Wavelet based (Wavelet) and FFT based (Welch) estimates of magnitude squared coherence (Cxy) and transfer function gain (Gxy) between systolic blood pressure (SBP) and systolic middle cerebral artery velocity ($MCAV_{sys}$) by transcranial doppler for very low frequency (VLF), low frequency (LF), and high frequency (HF) ranges during different levels of lower body negative and positive pressure and additional hypoxia. Confidence intervals (horizontal dotted lines) in Bland Altman plot were adjusted for repeated measurements using linear mixed model (Parker et al. 2016).

References:

Parker, Richard A., Christopher J. Weir, Noah Rubio, Roberto Rabinovich, Hilary Pinnock, Janet Hanley, Lucy McCloughan, et al. 2016. "Application of Mixed Effects Limits of Agreement in the Presence of Multiple Sources of Variability: Exemplar from the Comparison of Several Devices to Measure Respiratory Rate in COPD Patients." Edited by Hong-Long (James) Ji. *PLOS ONE* 11 (12): e0168321. https://doi.org/10.1371/journal.pone.0168321.
