# Supplementary material for: Continuous wavelet based transfer function analysis of cerebral autoregulation dynamics for neuromonitoring using near-infrared spectroscopy
Source: Front Physiol. 2025 Jun 18;16:1616125. doi: 10.3389/fphys.2025.1616125 (PMC12213380; doi:10.3389/fphys.2025.1616125)
Supplement: Supplementary file 4 [file DataSheet1.pdf]

Wavelet Transfer Function Estimation with Modified Cross Wavelet Function from Grinsted

André Diedrich and Surat Kulapatana, Vanderbilt University, Nashville, TN, USA

Abstract

Transfer function defines the relationship between the output response (Y) and to system input (X). Most software implementation of transfer function are FFT based algorithm with limited used for stationary data and low time resolution. Wavelet transform compromises between frequency and time resolution and it can be applied on non-stationary data. The original open-source cross wavelet function "xwt' from Grinsted calculate coherence and phase only. Estimates of transfer function are not available. We extend the cross wavelet function "xwt' from Grinsted et al to allow transfer function estimates based on wavelets similar to FFT based algorithm. The modified cross wavelet function "xwt_ext" implements correction of wavelet power bias and return of power and gain estimates.

Background

Transfer function defines the relationship between the output response (Y) and to system input (X). The transfer function (H) was calculated using cross and auto wavelet transform similar to FFT based algorithm (Zhang R et al. 1998):

$$H = \frac{W^{XY}}{W^{XX}}$$

where is W^{XY} is the cross wavelet transform of two time series X and Y which is defined as

$$W^{XY} = W^X * W^Y$$

and W^{XY} is the auto wavelet transform of time series X with

$$W^{XX} = W^X \star W^X$$

where * denotes complex conjugation.

Cross wavelet power (P^{XY}) and auto wavelet power (P^{XX}) is calculated as the absolute value or modulus.

$$P^{XY} = |W^{XY}|$$

$$P^{XX} = |W^{XX}|$$

The magnitude squared coherence (C^{XY}) function was calculated with

$$C^{XY} = \frac{|W^{XY}|^2}{W^{XX}\,W^{YY}}$$

A magnitude squared coherence value can be used to estimate the linear relations ship between X and Y. A value of 1 indicates a strong linear relationship. Zero value indicates no linear relationship. A squared coherence value threshold > 0.5 was defined as sufficient linear relationship to estimate transfer function gain.

Local phase (A^{XY}) between X and Y can be defined as the complex argument.

$$A^{XY} = arg(W^{XY})$$

The local transfer function gain G is defined as the magnitude and calculated as the square root of sum squared values of real and imaginary part of H.

$$G^{XY} = \sqrt{(Real(H))^2 + (Im(H))^2}$$

Implementation

The original open-source cross wavelet function "xwt' was extended to allow transfer function estimates based on wavelets similar to FFT based algorithm. The extended cross wavelet function "xwt_ext" implements correction of wavelet power bias and output power and gain estimates. Following changes in xwt function have been implemented:

We corrected for the wavelet power bias as proposed by Liu et al. 2007.

```
% AnDi added rectification of wavelet power and crosspectral power
% References: Liu Y et al. 2007; Veleda D et al. 2012
X = X ./sqrt(scale');
Y = Y ./sqrt(scale');
```

We applied smoothwavelet function for for auto W^{XX}, W^{YY} and cross wavelet W^{XY} before any further estimates of power, coherence, and phase.

```
% AnDi apply smoothwavelet and make sure Power is positve
sWxx = abs(smoothwavelet(sinv.*(abs(X).^2),dt,period,Args.Dj,scale));
sWyy = abs(smoothwavelet(sinv.*(abs(Y).^2),dt,period,Args.Dj,scale));

% AnDi smooth complex cross spectra
sWxy = smoothwavelet(sinv.*Wxy,dt,period,Args.Dj,scale);

% AnDi calulate R square (squared Coherence)
Rsq = abs(sWxy).^2./(sWxx.*sWyy);
```

We corrected sign of phase A^{XY} to currents standards where negative represents a leading phase which means input X leads Y .

```
% AnDi calculate angle Axy with negative angle means x leads y
Axy = -angle(sWxy);
```

We corrected by CDelta specific for Morlet mother wavelet as proposed by Torrens et al. 1998, Liu et al. 2007, and Veleda et al. 2012.

```
% AnDi added rectification for power see above
% we need also CDelta coefficient
% References: Liu Y et al. 2007; Veleda D et al. 2012

Cdelta = 0.776; % this is for the MORLET wavelet only
Pxx = Args.Dj*dt/Cdelta*sWxx;
Pyy = Args.Dj*dt/Cdelta*sWyy;
complex_Pxy = Args.Dj*dt/Cdelta.*sWxy;
```

We added estimation of gain G^{XY} and cross spectral power P^{XY}.

```
% AnDi added gain for output
Gxy = abs(complex_Pxy./Pxx);

% AnDi convert complex Pxy to Pxy
Pxy = abs(complex_Pxy);
```

We added estimates of power, phase, gain, and coherence to the output of function.

```
% AnDi output aditional variables Pxx, Pyy, Pxy, Axy, Gxy, Rsq
varargout={Wxy,period ,scale,coi,sig95, Pxx, Pyy, Pxy, Axy, Gxy, Rsq};
```

Further, we modified internal plot routine with plot using corrected phase (negative angle means x leads y) and plot frequency scale instead period scale. Please see complete code below.

Full Code of extended cross wavelet function "xwt_ext" to estimate Transfer function based on Grinsted "xwt" function

The complete modified code "xwt_ext.m" is listed below. Modifications are comment with % AnDi. Note that the function "xwt_ext.m" has to be placed in the same directory where "xwt.m" is stored. If function "xwt_ext.m" is used in a separate directory then you need to copy helper functions in "private" subdir and make them available in path. Otherwise the function "xwt_ext.m" can not access these helper functions in the private dir.

```
function varargout=xwt_ext(x,y,varargin)
%% modified by Andre Diedrich 2021-2025
%  for additional output of aWxy, Rsq, Pxx, Pyy, Pxy
% [Wxy, period, scale, coi, sig95, Pxx, Pyy, Pxy, Axy, Gxy, Rsq] =
xwt_ext(x,y,varargin);
%
%% Cross wavelet transform
% Creates a figure of cross wavelet power in units of
% normalized variance.
%
% USAGE: [Wxy,period,scale,coi,sig95]=xwt(x,y,[,settings])
%
% x & y: two time series
% Wxy: the cross wavelet transform of x against y
% period: a vector of "Fourier" periods associated with Wxy
% scale: a vector of wavelet scales associated with Wxy
% coi: the cone of influence
%
% Settings: Pad: pad the time series with zeros?
% .         Dj: Octaves per scale (default: '1/12')
% .         S0: Minimum scale
% .         J1: Total number of scales
% .         Mother: Mother wavelet (default 'morlet')
% .         MaxScale: An easier way of specifying J1
% .         MakeFigure: Make a figure or simply return the output.
% .         BlackandWhite: Create black and white figures
% .         AR1: the ar1 coefficients of the series
% .              (default='auto' using a naive ar1 estimator. See ar1nv.m)
% .         ArrowDensity (default: [30 30])
% .         ArrowSize (default: 1)
% .         ArrowHeadSize (default: 1)
%
% Settings can also be specified using abbreviations. e.g. ms=MaxScale.
% For detailed help on some parameters type help wavelet.
%
% Example:
%    t=1:200;
%    xwt(sin(t),sin(t.*cos(t*.01)),'ms',16)
%
% Phase arrows indicate the relative phase relationship between the series
% (pointing right: in-phase; left: anti-phase; down: series1 leading
% series2 by 90deg)
%
% Please acknowledge the use of this software in any publications:
%   "Crosswavelet and wavelet coherence software were provided by
%   A. Grinsted."
%
% (C) Aslak Grinsted 2002-2014
%
```

```
% http://www.glaciology.net/wavelet-coherence
% ------------------------------------------------------------------------
%The MIT License (MIT)
%
%Copyright (c) 2014 Aslak Grinsted
%
%Permission is hereby granted, free of charge, to any person obtaining a copy
%of this software and associated documentation files (the "Software"), to deal
%in the Software without restriction, including without limitation the rights
%to use, copy, modify, merge, publish, distribute, sublicense, and/or sell
%copies of the Software, and to permit persons to whom the Software is
%furnished to do so, subject to the following conditions:
%
%The above copyright notice and this permission notice shall be included in
%all copies or substantial portions of the Software.
%
%THE SOFTWARE IS PROVIDED "AS IS", WITHOUT WARRANTY OF ANY KIND, EXPRESS OR
%IMPLIED, INCLUDING BUT NOT LIMITED TO THE WARRANTIES OF MERCHANTABILITY,
%FITNESS FOR A PARTICULAR PURPOSE AND NONINFRINGEMENT. IN NO EVENT SHALL THE
%AUTHORS OR COPYRIGHT HOLDERS BE LIABLE FOR ANY CLAIM, DAMAGES OR OTHER
%LIABILITY, WHETHER IN AN ACTION OF CONTRACT, TORT OR OTHERWISE, ARISING FROM,
%OUT OF OR IN CONNECTION WITH THE SOFTWARE OR THE USE OR OTHER DEALINGS IN
%THE SOFTWARE.
%-------------------------------------------------------------------------

% AnDi
flg_contour = 0;

% ------validate and reformat timeseries.
[x,dt]=formatts(x);
[y,dty]=formatts(y);
if dt~=dty
    error('timestep must be equal between time series')
end
t=(max(x(1,1),y(1,1)):dt:min(x(end,1),y(end,1)))'; %common time period
if length(t)%3C4
    error('The two time series must overlap.')
end
n=length(t);

%----------default arguments for the wavelet transform-----------
Args=struct('Pad',1,...      % pad the time series with zeroes (recommended)
    'Dj',1/12, ...    % this will do 12 sub-octaves per octave
    'S0',2*dt,...    % this says start at a scale of 2 years
    'J1',[],...
    'Mother','Morlet', ...
    'MaxScale',[],...   %a more simple way to specify J1
    'MakeFigure',(nargout==0),...
    'HandleFigure',[],...
    'AR1','auto',...
```

```
    'ArrowDensity',[30 30],...
    'ArrowSize',1,...
    'ArrowHeadSize',1);
Args=parseArgs(varargin,Args,{'BlackandWhite'});
if isempty(Args.J1)
    if isempty(Args.MaxScale)
        Args.MaxScale=(n*.17)*2*dt; %auto maxscale
    end
    Args.J1=round(log2(Args.MaxScale/Args.S0)/Args.Dj);
end
ad=mean(Args.ArrowDensity);
Args.ArrowSize=Args.ArrowSize*30*.03/ad;
Args.ArrowHeadSize=Args.ArrowHeadSize*Args.ArrowSize*220;


if strcmpi(Args.AR1,'auto')
    Args.AR1=[ar1nv(x(:,2)) ar1nv(y(:,2))];
    if any(isnan(Args.AR1))
        error('Automatic AR1 estimation failed. Specify them manually (use the
arcov or arburg estimators).')
    end
end

%nx=size(x,1);
sigmax=std(x(:,2));

%ny=size(y,1);
sigmay=std(y(:,2));



%----------:::::::::::::--------- ANALYZE ----------::::::::::::------------

[X,period,scale,coix] =
wavelet(x(:,2),dt,Args.Pad,Args.Dj,Args.S0,Args.J1,Args.Mother);%#ok
[Y,period,scale,coiy] =
wavelet(y(:,2),dt,Args.Pad,Args.Dj,Args.S0,Args.J1,Args.Mother);

% AnDi added rectification of wavelet power and crosspectral power
% References: Liu Y et al. 2007; Veleda D et al. 2012
X = X ./sqrt(scale');
Y = Y ./sqrt(scale');

% truncate X,Y to common time interval (this is first done here so that the coi
is minimized)
dte=dt*.01; %to cricumvent round off errors with fractional timesteps
idx=find((x(:,1)%3E=(t(1)-dte))&(x(:,1)<=(t(end)+dte)));
X=X(:,idx);
coix=coix(idx);
```

```
idx=find((y(:,1)>=(t(1)-dte))&(y(:,1)<=(t(end)+dte)));
Y=Y(:,idx);
coiy=coiy(idx);

coi=min(coix,coiy);

% -------- Cross
Wxy=X.*conj(Y);



% AnDi uncommented following lines for export of Pxx,Pxy, Pyy, and Rsq
% --- was commmented in orig
% sinv=1./(scale');
% sinv=sinv(:,ones(1,size(Wxy,2)));
%
% sWxy=smoothwavelet(sinv.*Wxy,dt,period,dj,scale);
% Rsq=abs(sWxy).^2./(smoothwavelet(sinv.*
(abs(wave1).^2),dt,period,dj,scale).*smoothwavelet(sinv.*
(abs(wave2).^2),dt,period,dj,scale));
% freq = dt ./ period;
% --

sinv=1./(scale');
sinv=sinv(:,ones(1,size(Wxy,2)));

% AnDi apply smoothwavelet and make sure Power is positve
sWxx = abs(smoothwavelet(sinv.*(abs(X).^2),dt,period,Args.Dj,scale));
sWyy = abs(smoothwavelet(sinv.*(abs(Y).^2),dt,period,Args.Dj,scale));
% smooth complex cross spectra
sWxy = smoothwavelet(sinv.*Wxy,dt,period,Args.Dj,scale);

% AnDi calulate R square (squared Coherence)
Rsq  = abs(sWxy).^2./(sWxx.*sWyy);

% AnDi calculate angle Axy with negative angle means x leads y
Axy = -angle(sWxy);

% AnDi added rectification for power see above
% we need also CDelta coefficient
% References: Liu Y et al. 2007; Veleda D et al. 2012
Cdelta = 0.776;   % this is for the MORLET wavelet only
Pxx = Args.Dj*dt/Cdelta*sWxx;
Pyy = Args.Dj*dt/Cdelta*sWyy;
complex_Pxy = Args.Dj*dt/Cdelta.*sWxy;

% AnDi added gain for output
Gxy = abs(complex_Pxy./Pxx);
% AnDi convert complex Pxy to Pxy
Pxy = abs(complex_Pxy);
```

```
%---- Significance levels
%Pk1=fft_theor(freq,lag1_1);
%Pk2=fft_theor(freq,lag1_2);
Pkx=ar1spectrum(Args.AR1(1),period./dt);
Pky=ar1spectrum(Args.AR1(2),period./dt);


V=2;
Zv=3.9999;
signif=sigmax*sigmay*sqrt(Pkx.*Pky)*Zv/V;
sig95 = (signif')*(ones(1,n));  % expand signif --> (J+1)x(N) array
sig95 = abs(Wxy) ./ sig95;
if ~strcmpi(Args.Mother,'morlet')
    sig95(:)=nan;
end

% AnDi we create a new plot of all Pxx, Pyy, Pxy, Cxy, Gxy
% if Args.MakeFigure
%     Yticks = 2.^(fix(log2(min(period))):fix(log2(max(period))));
%
%     H=imagesc(t,log2(period),log2(abs(Wxy/(sigmax*sigmay))));%#ok
%     %logpow=log2(abs(Wxy/(sigmax*sigmay)));
%     %[c,H]=contourf(t,log2(period),logpow,[min(logpow(:)):.25:max(logpow(:))]);
%     %set(H,'linestyle','none')
%
%     clim=get(gca,'clim'); %center color limits around log2(1)=0
%     clim=[-1 1]*max(clim(2),3);
%     set(gca,'clim',clim)
%
%     HCB=colorbar;
%     set(HCB,'ytick',-7:7);
%     barylbls=rats(2.^(get(HCB,'ytick')'));
%     barylbls([1 end],:)=' ';
%     barylbls(:,all(barylbls==' ',1))=[];
%     set(HCB,'yticklabel',barylbls);
%
%     set(gca,'YLim',log2([min(period),max(period)]), ...
%         'YDir','reverse', ...
%         'YTick',log2(Yticks(:)), ...
%         'YTickLabel',num2str(Yticks'), ...
%         'layer','top')
%     %xlabel('Time')
%     ylabel('Period')
%     hold on
%
%     aWxy=angle(Wxy);
%
%     phs_dt=round(length(t)/Args.ArrowDensity(1));
tidx=max(floor(phs_dt/2),1):phs_dt:length(t);
%     phs_dp=round(length(period)/Args.ArrowDensity(2));
```

```
pidx=max(floor(phs_dp/2),1):phs_dp:length(period);
%
phaseplot(t(tidx),log2(period(pidx)),aWxy(pidx,tidx),Args.ArrowSize,Args.ArrowHea
dSize);
%
%     if strcmpi(Args.Mother,'morlet')
%         [c,h] = contour(t,log2(period),sig95,[1 1],'k');%#ok
%         set(h,'linewidth',2)
%     else
%         warning('XWT:sigLevelNotValid','XWT Significance level calculation is
only valid for morlet wavelet.')
%         %TODO: alternatively load from same file as wtc (needs to be coded!)
%     end
%     tt=[t([1 1])-dt*.5;t;t([end end])+dt*.5];
%     hcoi=fill(tt,log2([period([end 1]) coi period([1 end])]),'w');
%     set(hcoi,'alphadatamapping','direct','facealpha',.5)
%     hold off
% end


% AnDi new plot of all Pxx, Pyy, Pxy, Cxy, Gxy
if Args.MakeFigure

        hfig = Args.HandleFigure;
        if isempty(hfig)
            hfig = figure('name','WAVELET SPECTROGRAM');
        end

        %% plot log10 Pxx
        % AnDi use prctile to scale colors
        y = log10(Pxx+1E-32); % AnDi add 1E-32 to prevent log(0)
      ymax = prctile(y(:),99);
        ymin = prctile(y(:),1);
        ymean = mean(y(:));

        ax1 = subplot(3,2,1, 'Parent',hfig);
        Yticks = 2.^(fix(log2(min(period))):fix(log2(max(period))));


        Yticks = 2.^(fix(log2(min(period))):fix(log2(max(period))));
        H=imagesc(ax1, t,log2(period),y,[ymin,ymax]);

        HCB=colorbar(ax1, 'location','eastoutside');
        HCB.Ticks = linspace(ymin, ymax, 3);
        set(HCB,'yticklabel',num2cell([ymin, ymean, ymax]));

        set(ax1,'YLim',log2([2,max(period)]), ...
            'YDir','reverse', ...
            'YTick',log2(Yticks(:)), ...
            'YTickLabel',num2str(1./Yticks'), ...
```

```
    'layer','top')
xlabel(ax1,'Time (s)')
ylabel(ax1,'Frequency (Hz)')
title(ax1,'log10 Pxx');
hold on
tt=[t([1 1])-dt*.5;t;t([end end])+dt*.5];
hcoi=fill(tt,log2([period([end 1]) coi period([1 end])]),'w');
set(hcoi,'alphadatamapping','direct','facealpha',.3)
hold off

%% AnDi plot log10 Pyy
y = log10(Pyy+1E-32);
% AnDi use prctile to scale colors
ymax = prctile(y(:),99);
ymin = prctile(y(:),1);
ymean = mean(y(:));

ax2 = subplot(3,2,2, 'Parent',hfig);

Yticks = 2.^(fix(log2(min(period))):fix(log2(max(period))));
% AnDi use prctile to scale colors
H=imagesc(ax2, t,log2(period),y,[ymin,ymax]);

HCB=colorbar(ax2,'location','eastoutside');
% AnDi use prctile to scale colors
HCB.Ticks = linspace(ymin, ymax, 3);
set(HCB,'yticklabel',num2cell([ymin, ymean, ymax]));

set(ax2,'YLim',log2([2,max(period)]), ...
    'YDir','reverse', ...
    'YTick',log2(Yticks(:)), ...
    'YTickLabel',num2str(1./Yticks'), ...
    'layer','top')
xlabel(ax2,'Time (s)')
ylabel(ax2,'Frequency (Hz)')
title(ax2,'log10 Pyy');
hold on

tt=[t([1 1])-dt*.5;t;t([end end])+dt*.5];
hcoi=fill(tt,log2([period([end 1]) coi period([1 end])]),'w');
set(hcoi,'alphadatamapping','direct','facealpha',.3)
hold off




%% AnDi plot Pxy (modified source to use freq instead period)
y = log10(Pxy+1E-32);
% AnDi use prctile to scale colors
```

```
        ymax = prctile(y(:),99);
        ymin = prctile(y(:),1);
        ymean = mean(y(:));

        ax3 = subplot(3,2,3, 'Parent',hfig);
        Yticks = 2.^(fix(log2(min(period))):fix(log2(max(period))));
        % AnDi commented
        % H=imagesc(ax3, t,log2(period),log2(abs(Wxy/(sigmax*sigmay))));%#ok
        %logpow=log2(abs(Wxy/(sigmax*sigmay)));
        %[c,H]=contourf(t,log2(period),logpow,
[min(logpow(:)):.25:max(logpow(:))]);
        %set(H,'linestyle','none')

        %clim=get(ax3,'clim'); %center color limits around log2(1)=0
        %clim=[-1 1]*max(clim(2),3);
        %set(ax3,'clim',clim)

        % AnDi commented
        % HCB=colorbar(ax3, 'location','eastoutside');
        %set(HCB,'ytick',-7:7);
        %barylbls=rats(2.^(get(HCB,'ytick')'));
        %barylbls([1 end],:)=' ';
        %barylbls(:,all(barylbls==' ',1))=[];
        %set(HCB,'yticklabel',barylbls);

        % AnDi new color scaling using prctile
        H=imagesc(ax3, t,log2(period),y,[ymin,ymax]);
        HCB=colorbar(ax3,'location','eastoutside');
        HCB.Ticks = linspace(ymin, ymax, 3);
        set(HCB,'yticklabel',num2cell([ymin, ymean, ymax]));


        set(ax3,'YLim',log2([2,max(period)]), ...
            'YDir','reverse', ...
            'YTick',log2(Yticks(:)), ...
            'YTickLabel',num2str(1./Yticks'), ...
            'layer','top')
        xlabel(ax3, 'Time (s)')
        ylabel(ax3, 'Frequency (Hz)')
        title('log10 Pxy')
        hold on

        % AnDi use corrected angles (-angle(Wxy)
        % negative values means x is leading (y lags x)
        % aWxy=angle(Wxy);
        % aWxy = Axy;
        %phs_dt=round(length(t)/Args.ArrowDensity(1));
tidx=max(floor(phs_dt/2),1):phs_dt:length(t);
        % phs_dp=round(length(period)/Args.ArrowDensity(2));
pidx=max(floor(phs_dp/2),1):phs_dp:length(period);
```

```
        % phaseplot_ext(ax3,
t(tidx),log2(period(pidx)),aWxy(pidx,tidx),Args.ArrowSize,Args.ArrowHeadSize);

        if strcmpi(Args.Mother,'morlet') & flg_contour == 1
            if isreal(sig95)
                [c,h] = contour(ax3, t,log2(period),sig95,[1 1],'k');%#ok
                set(h,'linewidth',2)
            end
        else
            warning('XWT:sigLevelNotValid','XWT Significance level calculation is
only valid for morlet wavelet.')
            %TODO: alternatively load from same file as wtc (needs to be coded!)
        end
        tt=[t([1 1])-dt*.5;t;t([end end])+dt*.5];
        hcoi=fill(tt,log2([period([end 1]) coi period([1 end])]),'w');
        set(hcoi,'alphadatamapping','direct','facealpha',.3)
        hold off


        %% AnDi plot squared coherence
        y = sqrt(Rsq);
        ax4 = subplot(3,2,4, 'Parent',hfig);
        Yticks = 2.^(fix(log2(min(period))):fix(log2(max(period))));
        H=imagesc(ax4, t,log2(period),y,[0,1]);

        HCB=colorbar(ax4, 'location','eastoutside');
        HCB.Ticks = linspace(0, 1, 5);
        set(HCB,'yticklabel',num2cell([0,0.25,0.5,0.75,1]));

        set(ax4,'YLim',log2([2,max(period)]), ...
            'YDir','reverse', ...
            'YTick',log2(Yticks(:)), ...
            'YTickLabel',num2str(1./Yticks'), ...
            'layer','top')
        xlabel(ax4, 'Time (s)')
        ylabel(ax4, 'Frequency (Hz)')
        title (ax4, 'Cxy');
        hold on

        % AnDi use corrected angles
        % negative values means x is leading (y lags x)
        % orignal uses angle(Wxy);
        % new uses    -angle(Wxy)
        aWxy = Axy;

        phs_dt=round(length(t)/Args.ArrowDensity(1));
tidx=max(floor(phs_dt/2),1):phs_dt:length(t);
        phs_dp=round(length(period)/Args.ArrowDensity(2));
pidx=max(floor(phs_dp/2),1):phs_dp:length(period);
```

```
phaseplot_ext(ax4,t(tidx),log2(period(pidx)),aWxy(pidx,tidx),Args.ArrowSize,Args.
ArrowHeadSize);

        if strcmpi(Args.Mother,'morlet') & flg_contour  == 1
            if isreal(sig95)
                [c,h] = contour(ax4, t,log2(period),sig95,[1 1],'k');%#ok
                set(h,'linewidth',2)
            end
        else
            warning('XWT:sigLevelNotValid','XWT Significance level calculation is
only valid for morlet wavelet.')
            %TODO: alternatively load from same file as wtc (needs to be coded!)
        end
        tt=[t([1 1])-dt*.5;t;t([end end])+dt*.5];
        hcoi=fill(tt,log2([period([end 1]) coi period([1 end])]),'w');
        set(hcoi,'alphadatamapping','direct','facealpha',.3)
        hold off

        % plot Gain in Decibel
        y = 20*log10(Gxy+1E-32); % in decibel from power ratio
        ymax = prctile(y(:),99);
        ymin = prctile(y(:),1);
        ymean = mean(y(:));

        ax5 = subplot(3,2,5,'Parent',hfig);

        Yticks = 2.^(fix(log2(min(period))):fix(log2(max(period))));
        % AnDi scale color with prctile in Decibel
        H=imagesc(ax5, t,log2(period),y,[ymin,ymax]);

        HCB=colorbar(ax5, 'location','eastoutside');
        HCB.Ticks = linspace(ymin, ymax, 3);
        % AnDi scale color prctile in Decibel,
        % +3 Decibel is equal gain 2
        %  0 Decibel is equal gain 1
        % -3 Decibel is equal gain 2
        set(HCB,'yticklabel',num2cell([ymin, 0, ymax]));

        set(ax5, 'YLim',log2([2,max(period)]), ...
            'YDir','reverse', ...
            'YTick',log2(Yticks(:)), ...
            'YTickLabel',num2str(1./Yticks'), ...
            'layer','top')
        xlabel(ax5, 'Time (s)')
        ylabel(ax5, 'Frequency (Hz)')
        hold on
        title(ax5, 'Gxy (dB)');

        % AnDi use corrected angles (-angle(Wxy)
        % negative values means x is leading (y lags x)
```

```
        % aWxy=angle(Wxy);
        % aWxy = Axy;
        % phs_dt=round(length(t)/Args.ArrowDensity(1));
tidx=max(floor(phs_dt/2),1):phs_dt:length(t);
        % phs_dp=round(length(period)/Args.ArrowDensity(2));
pidx=max(floor(phs_dp/2),1):phs_dp:length(period);
        % phaseplot_ext(ax5,
t(tidx),log2(period(pidx)),aWxy(pidx,tidx),Args.ArrowSize,Args.ArrowHeadSize);

        if strcmpi(Args.Mother,'morlet') & flg_contour == 1
            if isreal(sig95)
                [c,h] = contour(ax5, t,log2(period),sig95,[1 1],'k');%#ok
                set(h,'linewidth',2)
            end
        else
            warning('XWT:sigLevelNotValid','XWT Significance level calculation is
only valid for morlet wavelet.')
            %TODO: alternatively load from same file as wtc (needs to be coded!)
        end
        tt=[t([1 1])-dt*.5;t;t([end end])+dt*.5];
        hcoi=fill(tt,log2([period([end 1]) coi period([1 end])]),'w');
        set(hcoi,'alphadatamapping','direct','facealpha',.3)
        hold off

end


% AnDi output aditional variables Pxx, Pyy, Pxy, Axy, Gxy, Rsq
% varargout={Wxy,period ,scale,coi,sig95}
varargout={Wxy,period ,scale,coi,sig95, Pxx, Pyy, Pxy, Axy, Gxy, Rsq};
varargout=varargout(1:nargout);>)
```

References

Grinsted, A., J. C. Moore, and S. Jevrejeva. 2004. “Application of the Cross Wavelet Transform and Wavelet Coherence to Geophysical Time Series.” Nonlinear Processes in Geophysics 11 (5/6): 561–66. https://doi.org/10.5194/npg-11-561-2004.

Torrence, Christopher, and Gilbert P. Compo. 1998. “A Practical Guide to Wavelet Analysis.” *Bulletin of the American Meteorological Society* 79 (1): 61–78. https://doi.org/10.1175/1520-0477(1998)079<0061:APGTWA>2.0.CO;2.

Liu, Yonggang, X. San Liang, and Robert H. Weisberg. 2007. “Rectification of the Bias in the Wavelet Power Spectrum.” *Journal of Atmospheric and Oceanic Technology* 24 (12): 2093–2102. https://doi.org/10.1175/2007JTECHO511.1.

Veleda, Doris, Raul Montagne, and Moacyr Araujo. 2012. “Cross-Wavelet Bias Corrected by Normalizing Scales.” Journal of Atmospheric and Oceanic Technology_ 29 (9): 1401–8. https://doi.org/10.1175/JTECH-D-11-00140.1.

Zhang, Rong, Julie H. Zuckerman, Cole A. Giller, and Benjamin D. Levine. “Transfer Function Analysis of Dynamic Cerebral Autoregulation in Humans.” *American Journal of Physiology-Heart and Circulatory Physiology* 274, no. 1 (January 1, 1998): H233–41. https://doi.org/10.1152/ajpheart.1998.274.1.H233
